# Decomposition of Selenourea in Various Solvents: Red versus Gray Selenium in the Synthesis of Iron Selenide Nanoparticles

**DOI:** 10.1021/acs.chemmater.4c03430

**Published:** 2025-06-10

**Authors:** Andrey A. Shults, Alexandra C. Koziel, Joshua D. Caldwell, Janet E. Macdonald

**Affiliations:** 1 Department of Chemistry, 5718 Vanderbilt University, Nashville, Tennessee 37235, United States; 2 Vanderbilt Institute for Nanoscale Science and Engineering, Nashville, Tennessee 37235, United States; 3 Department of Mechanical Engineering, Vanderbilt University, Nashville, Tennessee 37235, United States

## Abstract

Selenourea is a useful reagent for the synthesis of metal chalcogenides. Its low decomposition temperature allows it to bypass the usual harsh conditions used in nanoparticle synthesis. Here, we show that selenourea decomposes differently based on its chemical environment, a phenomenon that can be used for the phase control of iron selenide nanoparticles. Two solvents (oleylamine and oleic acid) were tested for their interactions with selenourea to control the phase of iron selenides as they are ubiquitous in nanocrystal synthesis. It was found that in the presence of oleylamine, selenourea decomposes into red selenium resulting in the formation of Fe_7_Se_8_. When combined with oleic acid, selenourea decomposes into gray selenium, resulting in the formation of FeSe_2_. In this report, allotropes have been identified as phase-determining intermediates in nanocrystal synthesis.

## Graphical abstract

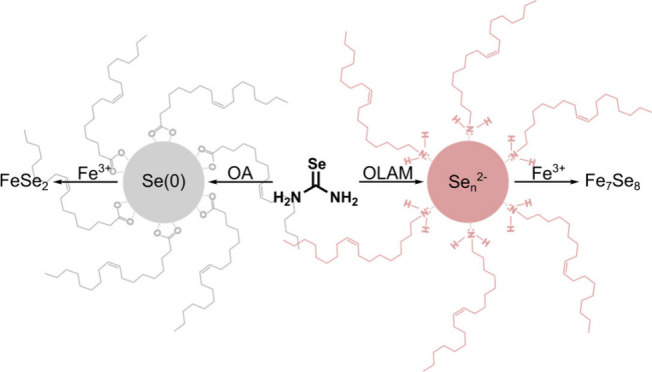

## Introduction

Selenourea is an inorganic reagent that can be used for the low-temperature synthesis of metal selenide nanoparticles, yet not much is known about its decomposition in long chain organic solvents/ligands such as oleic acid (OA) and oleylamine (OLAM). To achieve rational and repeatable synthesis of metal chalcogenides, it is imperative we understand the precursor chemistry involved in the formation of nanoparticles.^1–5^ By doing so, it would be possible to fully utilize the electronic, optical, magnetic, and catalytic properties of every phase of a metal chalcogenide family.^6–10^

Just like its sulfur analogue (thiourea), selenourea became popular for metal selenide syntheses because it is stable at room temperature, does not decompose in light, and readily reacts with metals at temperatures as low as 100 °C. Additionally, the decomposition rate and temperature of selenourea can be affected by N-substitution with various organic groups.^11^ Selenourea and its derivatives are convenient reagents for the colloidal synthesis of metal selenides.

Iron selenides are interesting synthetic targets due to the quantity of known crystalline structures. In the iron selenide family, there are a total of six known phases: Fe_3_Se_4_, FeSe_2_, Fe_1–*x*_Se (α-FeSe), Fe_1+*x*_Se (β-FeSe), and Fe_7_Se_8_ (3C and 4C) (Table 1). Fe_3_Se_4_ has a spinel structure (Fe^2+^Fe^3+^_2_Se_4_), where the Se^2–^ takes on a face-centered-cubic arrangement, and the cations are mixed between tetrahedral and octahedral sites containing both Fe^2+^ and Fe^3+^.^12^ FeSe_2_ has an orthorhombic marcasite structure, which is comprised of a hexagonal close packed (hcp) arrangement of Se_2_^2–^ dimers with Fe^2+^ filling every octahedral site.^12^ There is some confusion in the literature between α- and β-FeSe. Here, α-FeSe is defined as NiAs-type, and β-FeSe is PbO-type. Fe_7_Se_8_ is based on NiAs-type FeSe with a cation-deficient structure. The ordering of the cation vacancies between every second close packed anion layer gives two main superstructures: orthorhombic 4C and trigonal 3C.^13^ While the arrangements of the vacancies in the AB planes are identical, the stacking sequence of the planes and their number lead to these specific structures.^14^

**Table 1:** Experimentally Known Phases of the Iron Selenide Family

common name	stoichiometry	crystal structure	space group	lattice parameters (Å)	ref
	Fe_3_Se_4_	Monoclinic	*I*12/*m*1	*a* = 6.20, *b* = 3.53, *c* = 11.26	Berodias et al.^15^
Ferroselite	FeSe_2_	Orthorhombic marcasite	*Pnnm*	*a* = 4.80, *b* = 5.78, *c* = 3.59	Söchtig et al.^16^
α-FeSe	Fe_1–*x*_Se	Hexagonal NiAs	*P*63/*mmc*	*a* = *b* = 3.76, *c* = 5.96	Koz et al.^17^
β-FeSe	Fe_1+*x*_Se	Tetragonal PbO	*P*4/*nmm*	*a* = *b* = 3.79, *c* = 5.51	Kovnir et al.^18^
3C-Fe_7_Se_8_	Fe_7_Se_8_	NiAs superstructure (trigonal)	*P*3_1_21	*a* = *b* = 7.23, *c* = 17.60	Mozgovykh et al.^13^
4C-Fe_7_Se_8_	Fe_7_Se_8_	Pseudo-NiAs superstructure (orthorhombic)	*C*222	*a* = 12.53, *b* = 7.23, *c* = 23.54	Okazaki et al.^19^

The iron selenides present an impressive array of magnetic and optoelectronic properties. Both Fe_3_Se_4_ and Fe_7_Se_8_ exhibit above room-temperature ferrimagnetic ordering with Curie temperatures of *T*_C_ = 314 K and *T*_C_ = 450 K, respectively.^20,21^ Fe_3_Se_4_ exhibits giant coercivity of 40 kOe at 10 K, which has been attributed to the material’s large magnetocrystalline anisotropy.^22^ PbO-type FeSe and FeSe_2_ have strong photoluminescence properties; PbO-type FeSe was found to have a quantum yield of 20% while FeSe_2_ was 16%.^23^ PbO-type FeSe also shows superconducting properties below a critical temperature of 8 K at standard pressure.^24^

The reported syntheses of iron selenides often require high temperatures, even in colloidal syntheses. Both FeSe_2_ and Fe_7_Se_8_ have been previously reported in literature. Fe_7_Se_8_ has been achieved using high temperature methods like the Bridgman method that involves heating to temperatures of around 1000 °C.^13,25^ FeSe_2_ has been previously synthesized using hydrothermal, tube furnace, and solvothermal methods.^12,26^ Some of the more common precursors for these syntheses involved elemental selenium for the selenium precursor; and iron halides, iron sulfates, and iron nitrates for the iron precursor. The synthesis temperature ranged from 140 to 200 °C for hydrothermal methods, from 350 to 500 °C for tube furnace methods, and from 140 to 330 °C for solvothermal methods. With the use of selenourea, there is a higher potential to prepare these materials in much more moderate temperature ranges.

Long chain carboxylic acids and amines are ubiquitous to nanocrystalline synthesis. These ligands have high-boiling points (>300 °C), can stabilize the surfaces of growing nanocrystals, and have long been recognized for providing size and shape control.^3,27,28^ Ligands also influence the crystalline phase that forms, and due to their frequent use, it is essential that we understand their effect. Acid and amine ligands are also known to act as reagents and influence the nanoparticle phase. For instance, Hollingsworth et al. showed that when combining OLAM with a single source precursor, nickel dithiocarbamate, the OLAM chemically changed the nickel environment.^1^ The OLAM was able to perform a slow amide-exchange with the complex, assisting with its thermal decomposition and allowing for low temperature synthesis of NiS nanoparticles. Amines in general can also react with thioacetamide, a very common sulfur source in nanosynthesis, by nucleophilic substitution (S_N_2) and release H_2_S, which can act as an active sulfur source.^29,30^ Finally, OLAM can form an amine–polysulfide complex with elemental sulfur (S_8_), which upon heating to reaction temperature can react with excess amine and result in the formation of H_2_S, an active sulfur source in the metal sulfide synthesis.^3^ It is not known if any or all of this chemistry is relevant to the selenium system.

Similarly, OA can have unexpected effects on the molecular chemistry in nanocrystal synthesis. Our group has previously shown that OA can push thiourea to isomerize into ammonium thiocyanate, a more reluctant sulfur source.^31^ With an increase in the concentration of OA, more sulfur-poor nanoparticles would form. Substituted thioureas follow a similar pathway, but they instead isomerize to isothiocyanates and primary amines.^30,32^ When a carboxylate is added to the mix, carbonyl sulfide forms and acts as the primary sulfur source instead of the substituted thiourea which can normally directly bind to the metal source.^33–35^ Again, it is not clear whether this chemistry with OA is relevant to selenourea.

While one might imagine that the well-studied chemistry of thiourea can be translated to selenourea, there are significant differences. Compared to thiourea, selenourea shows a longer and weaker carbon–chalcogen bond and a lower oxidation potential (thiourea *E*_ox_ = 1.07 eV, selenourea *E*_ox_ = 0.50 eV).^36^ Furthermore, if elemental selenium is an intermediate, then there are several known allotropes. The most common, crystalline gray selenium, Se(0), contains long one-dimensional chains of Se.^37^ In contrast, crystalline red selenium, Se_n_^2–^, has three allotropes consisting of monoclinic Se_8_ rings of various symmetries.^37^ Additionally, amorphous selenium is another allotrope that can be red. The structure of this allotrope remains under debate regarding the ratio of selenium rings versus chains contained within the structure.^38^ There are also several known structures of crystalline polyselenides, where negatively charged Se_n_ rings are counterbalanced with cations such as Cs^+^.^39^ Selenourea may have an entirely unique chemistry compared with thiourea in the context of nanocrystal synthesis.

Herein, we examine the decomposition pathways of selenourea in the presence of OLAM and OA for the formation of iron selenides. In OLAM, Fe_7_Se_8_ nanocrystals result, whereas in OA, the product is FeSe_2_. Using spectroscopic techniques of nuclear magnetic resonance (^1^H and ^13^C NMR) and gas phase Fourier transform infrared (FTIR) spectroscopy, we follow the fate of selenourea through the reactions and find that the active selenium source is Se(0) from the thermal decomposition of selenourea. In OLAM, red selenium is the major selenium source, while gray selenium is the primary source when OA is used. It is these different intermediate allotropes of selenium that lead to the phase control described here.

## Results and Discussion

To first demonstrate the effect of ligand choice on the nanocrystal phase when selenourea was used as a selenium source, iron selenides were synthesized at variable ratios of OLAM to OA and different temperatures (Figure 1A). Characterization with powder X-ray diffraction (pXRD) (Figure S1) showed a clear difference in the resulting iron selenide phase depending on the ligand employed (Figure 1B). In OA, the selenium rich phase (FeSe_2_) formed, whereas when OLAM was used as a solvent, the selenium poor phase (3C-Fe_7_Se_8_) formed. Mixtures of OA and OLAM in the reactions gave a mixture of FeSe_2_ and Fe_7_Se_8_. At lower temperatures (100, 120 °C) Fe_7_Se_8_ dominated, but at higher temperatures (140, 200 °C) FeSe_2_ was preferred. 3C-Fe_7_Se_8_ is similar in structure to NiAs-type Fe_1–*x*_Se but has layered vacancies which give a distinctive reflection at 2θ ∼ 15° in the pXRD (Figure S1).^13^

**Figure 1:**
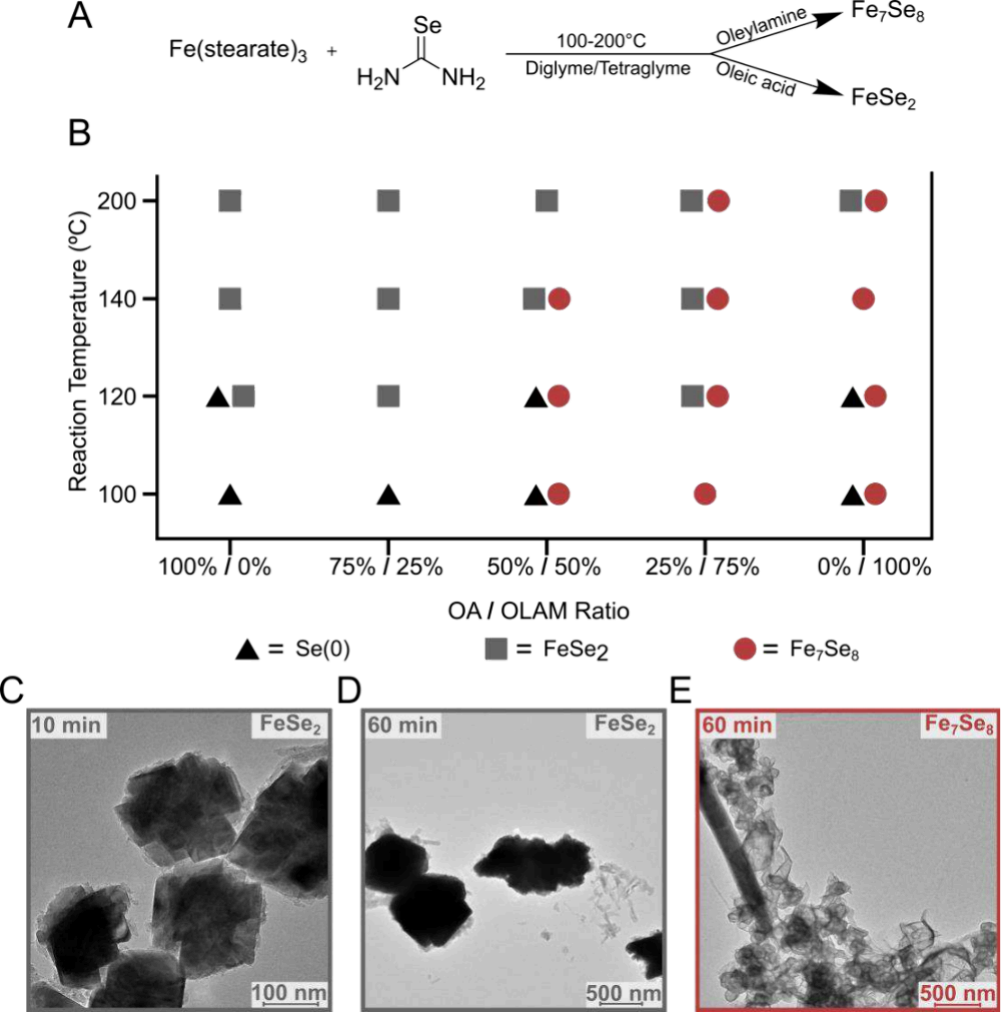
(A) General reaction scheme for the synthesis of iron selenides in OA and OLAM. (B) Phase composition trends of iron selenides synthesized using varying ratios of OA and OLAM and reaction temperatures. Black triangle represents Se(0), gray square represents FeSe_2_, and brick red circle represents Fe_7_Se_8_. Bright field TEM of (C) congregated cubes of FeSe_2_ produced in OA at 10 min of synthesis time and 140 °C, (D) congregated cubes of FeSe_2_ produced in OA at 60 min of synthesis time and 140 °C, and (E) Fe_7_Se_8_ layered structures produced in OLAM at 60 min of synthesis time and 140 °C. A rod of selenium is also imaged along with the Fe_7_Se_8_ particles.

Gray Se(0) was also an observed product by pXRD in every ligand mixture at low temperatures of 100 °C. Gray Se(0) was occasionally and inconsistently identified in reactions at higher temperatures, and we concluded the variation depends on effectiveness of the cleaning process and that more of the selenium has been consumed at the elevated temperatures. It is likely, therefore, that Se(0) is an intermediate of the reaction between selenourea and iron­(III) stearate to form iron selenides.

Transmission electron microscopy (TEM) and high-angle annular dark-field scanning transmission electron microscopy coupled with energy dispersive X-ray spectroscopy (HAADF-STEM-EDS) images were collected to confirm the morphology and elemental distributions in the resulting nanocrystals (Figures 1 and S16–S18). For reaction conditions of TEM analyzed samples, 140 °C and 100% OA or OLAM were picked due to pXRD patterns sowing phase purity. FeSe_2_ particles resembled large congregations of faceted crystals, which grew with time. At 10 min, their size was around 100 nm (Figure 1C), and after an hour they grew larger than 500 nm (Figure 1D). Looking closely, each particle was still comprised of smaller structures, a morphology that was easily detected on the HAADF images. In the 10 min sample, a selenium particle was also imaged with small FeSe_2_ nanoparticles growing on its sides. After an hour, no pure selenium particles were detected. In contrast, the Fe_7_Se_8_ formed as spheroids of crumbled sheets with a size of around 500 nm (Figure 1E). The selenium impurity imaged in this case was long rods. To understand the source of the ligand-mediated phase control of iron selenides, we aimed to follow the path of the selenourea precursor decomposition in OLAM and OA. To do so, we used gas-phase FTIR and ^1^H and ^13^C NMR of the decomposition byproducts along with multiple controls.

The gaseous products of the reaction between selenourea and OLAM were monitored between 25 and 200 °C using *in situ* gas FTIR spectroscopy (Figure 2A, full spectrum Figure S7). In all spectra, both water and ammonia were identified, with the amount of ammonia increasing with increasing reaction temperature (Figure S11). Selenourea was clearly reacting, but no carbon or selenium-based gases were seen at any of the temperatures indicating the carbon and selenium byproducts remained in solution. The solution also turned red, suggesting the formation of red selenium.

**Figure 2:**
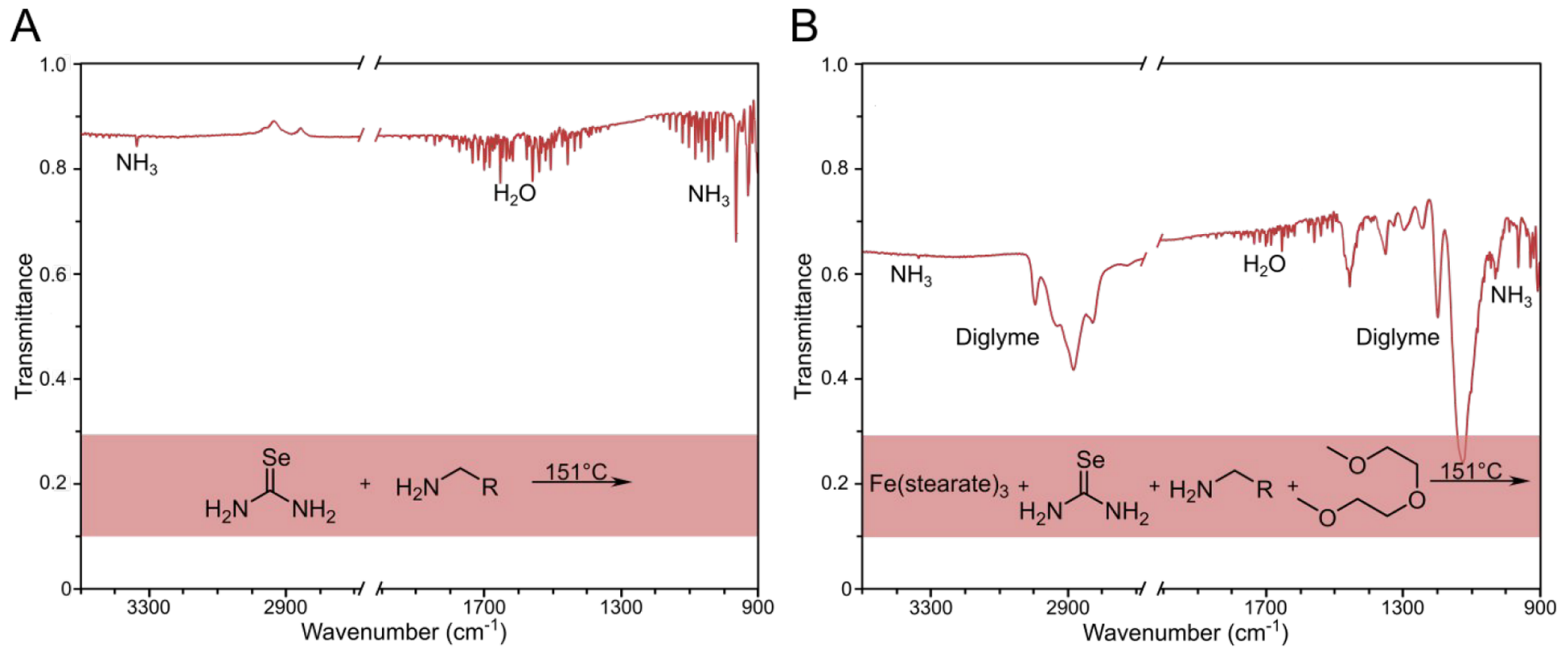
Gas FTIR of thermal decomposition products of (A) selenourea (0.75 mmol) and OLAM (15.2 mmol) at 151 °C and (B) iron­(III) stearate (0.25 mmol), selenourea (0.75 mmol), OLAM (15.2 mmol), and diglyme (34.9 mmol) at 151 °C.

When selenourea and OLAM were reacted in diglyme with iron­(III) stearate (Figure 2B), water and ammonia were again detected, along with the solvent diglyme (bp 162 °C). Again, no carbon- or selenium-based gaseous decomposition products were identified. This time, the solution turned black, indicating the formation of an iron selenide.

A very different set of gaseous decomposition products was seen when OA was used instead of OLAM. When selenourea was heated with OA, only water was identified at 107 °C (Figure 3A, full spectrum Figure S8). At 147 °C and above, CO_2_ was identified, which increased in intensity with the reaction temperature. A small amount of CO was also present at the highest temperatures of 204 °C. Both can be ascribed to the decarboxylation of OA. The remaining solution turned black, suggesting the formation of Se(0).

**Figure 3:**
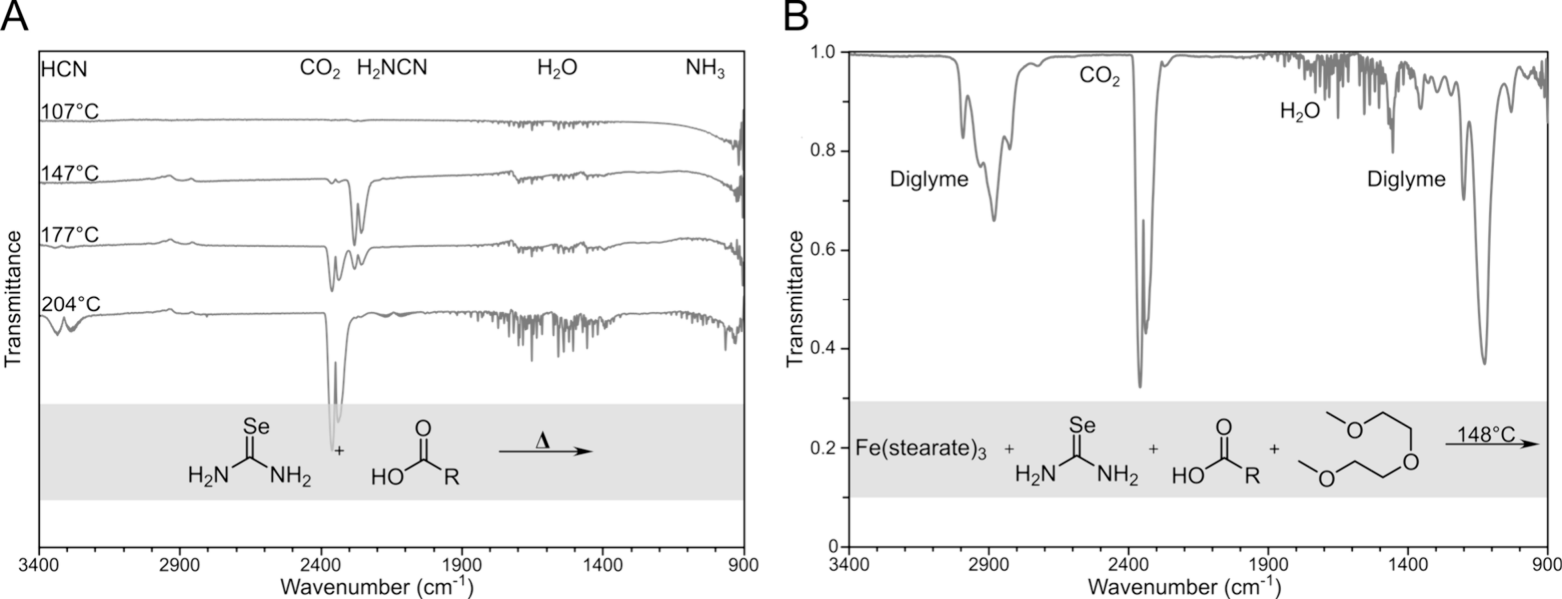
Gas FTIR of thermal decomposition products of (A) selenourea (0.75 mmol) in OA (15.8 mmol) at a range of temperatures from 107 to 204 °C and (B) iron­(III) stearate (0.25 mmol), selenourea (0.75 mmol), OA (15.8 mmol), and diglyme (34.9 mmol) at 148 °C.

In addition to CO_2_, in the presence of OA, selenourea decomposed to give gaseous C and N species, unlike when it was reacted in OLAM. At 147 °C, a new doublet of peaks was found at around 2281 and 2269 cm^–1^ and can be attributed to a nitrile group (C≡N), most likely from cyanamide (H_2_NCN).^40^ At higher temperatures (>150 °C), the cyanamide peak decreased in intensity, and a new set of peaks formed: a doublet at 3284 and 3336 cm^–1^ indicating hydrogen cyanide (HCN)^41^ and a collection of peaks between 1177 and 910 cm^–1^ representing ammonia (NH_3_).^42^ Together it suggests selenourea decomposes in OA to give cyanamide, which at temperatures above 177 °C further decomposes to hydrogen cyanide and ammonia.^41,42^

When selenourea was reacted with iron­(III) stearate in the presence of diglyme and OA, only two gases were identified: CO_2_ (decarboxylation) and diglyme (evaporation) (Figure 3B). The process that produces gaseous cyanamide was circumvented by the presence of Fe­(III) and the formation of FeSe_2_. To confirm that diglyme is not reacting with cyanamide, the reagents of selenourea, OA, and diglyme were heated together and analyzed with gas phase FTIR (Figure S9). The trend in the formation of cyanamide and CO_2_ does not change.

Because selenium-based species were not detected in the gas phase, we turned to ^1^H NMR to observe any remaining decomposition products of selenourea (Figures 4 and S19–S28). Examining the temperature studies of the reaction between selenourea and OLAM, the spectrum remained unchanged until around 160 °C (Figure 4A). At this point, the solution was red and cloudy, and multiple broad singlets started appearing in the ^1^H NMR spectrum at δ = 0.59, 1.00, 1.70, 2.30, and 5.01 ppm. The positions of the ^13^C NMR signals did not change, suggesting that these shifting ^1^H NMR signals were from NH species in differing chemical environments, including amine and ammonium species, with various counterions. One interpretation is that ammonium groups were paired with various polyselenide units, Se_n_^2–^, resulting in multiple broad singlets, each with its own characteristic chemical shift. The result of isolated speciation of polyselenide, oleylammonium, or ammonium polyselenide contrasts with ^1^H NMR studies of OLAM and oligomeric sulfur, which produced only one N–H resonance, due to fast exchange on the NMR time scale.^3^

**Figure 4:**
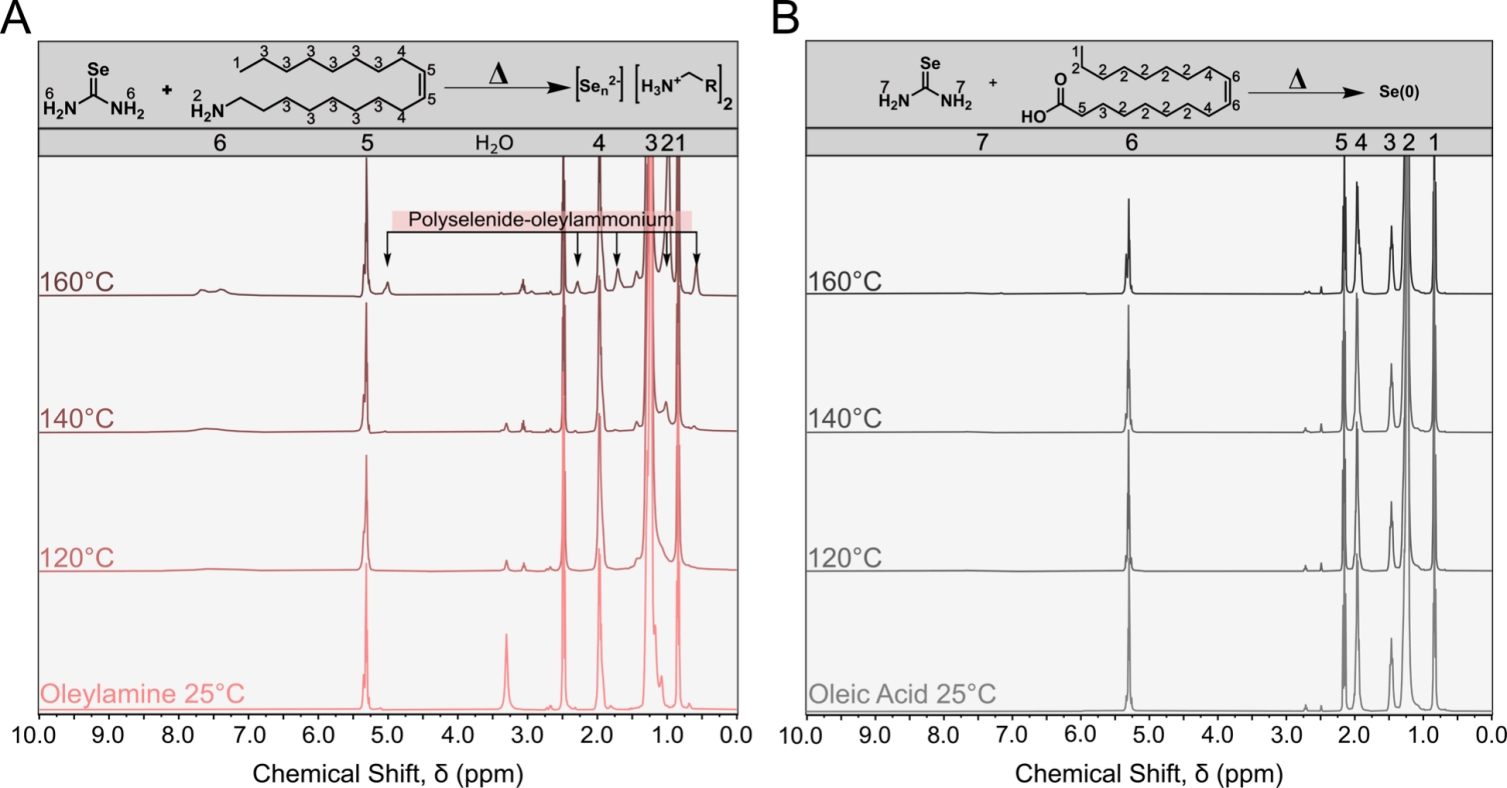
^1^H NMR of the reactions between (A) selenourea (0.1 mmol) and OLAM (0.1 mmol) and (B) selenourea (0.1 mmol) and OA (0.1 mmol) at various temperatures in 600 μL of DMSO-*d*_6_. The peaks have been labeled to their corresponding hydrogen using a numbered system of 1 through 7 and positioned between the reaction schemes and the spectra.

The red color of the precipitate produced from the control reaction of selenourea, OLAM, and diglyme is characteristic of red selenium. After an hour of reacting, the selenourea decomposed into a mixture of red and gray selenium, as characterized by pXRD (Figure S4). The low intensity peak at around 20° is difficult to identify, whether it belongs to red selenium or a small portion of unreacted selenourea. pXRD of the red product on day 0 gave multiple sharp peaks below 2θ = 20°, a region where molecular crystals diffract due to their large unit cells. This pattern also contained minor peaks of gray Se(0), which increased in intensity over hours and days. Over 1 day, the brick red powder started to turn into a gray powder indicating the formation of gray selenium. After several days, the sample turned completely gray. The presence of red selenium is not inconsistent with the formation of polyselenides, as there are several known structures of crystalline polyselenides, where negatively charged Se_n_ rings are counterbalanced with cations such as Cs^+^.^39^

The above observations suggest that selenourea is not the active selenium reagent in the formation of Fe_7_Se_8_ in OLAM. Instead, red selenium forms *in situ* and is the active reagent. To obtain red selenium as an intermediate, OLAM must have acted as a reducing agent to the selenourea.

To provide further evidence that red selenium is indeed the direct source of selenium monomers in the synthesis of Fe_7_Se_8_, a mixture of selenourea, OLAM, and diglyme was reacted at 140 °C for 1 h to preform red selenium. Iron­(III) stearate was injected into the solution, and the resulting phase synthesized was Fe_7_Se_8_, the same phase obtained when selenourea is injected into iron­(III) stearate dissolved in OLAM (Figure S2).

The behavior of selenourea in OA was much simpler than that in OLAM. When OA was reacted with selenourea at various temperatures, no changes in the ^1^H NMR spectra were observed (Figure 4B). In contrast to OLAM, OA does not react with selenourea to form red selenium. This is further confirmed by the color and crystal structure of the resulting selenium powder. pXRD of the product showed only Se(0), and the initial gray color of the powder remained unchanged with respect to time. Once again, to test that gray selenium is the primary source of selenium when OA is used, iron­(III) stearate was injected into a mixture of selenourea, OA, and diglyme that was reacted at 140 °C for 1 h. The resulting phase synthesized was FeSe_2_, the same phase obtained when selenourea is injected into iron­(III) stearate dissolved in OA (Figure S3).

To demonstrate the importance of elemental selenium as a reactive intermediate, aliquot studies were performed for the reaction with 100% OLAM at 140 °C (Figure S6). One minute after the injection, only amorphous species were identified by pXRD with no evidence of iron selenide nanoparticles. Fe_7_Se_8_ began to form at as early as 5 min along with gray selenium, yet no FeSe_2_ was detected. Both Fe_7_Se_8_ and Se(0) continued increasing in intensity until 180 min of reaction time, when Se(0) signal disappeared, and two additional reflections at 34.9 and 36.2° formed indicating slow formation of FeSe_2_. These findings confirm that gray selenium is necessary for the formation of FeSe_2_ and acts as a direct source of selenium.

To confirm that elemental selenium is not only the active intermediate but also a phase-determining intermediate in the formation of iron selenides, reactions in a 50/50 OLAM/OA solvent mixture were examined more closely (Figure 5). A series of experiments were performed with identical solvent conditions but with the order of operations changed to cause the preformation of red or gray selenium before reaction with the iron. As the control, when a diglyme solution of selenourea is injected into a 50/50 mixture of OA/OLAM and iron­(III) stearate at 140 °C (1 h), the product is a mixture of the two iron selenides. In contrast, if the selenourea is allowed to react in OA/diglyme for 1 h, producing a gray solution of selenium, and then a solution of iron­(III) stearate in OLAM is injected, the product is almost exclusively FeSe_2_. If instead the selenourea is reacted in OLAM/diglyme producing a red solution of selenium (which is likely a mix of red and gray (see Figure S4), followed by injection of iron­(III) stearate in OA, the product still contains significant amounts of Fe_7_Se_8_. Similarly if the same procedure is performed at 120 °C, the control has a mixture of the two phases, the one with preformed gray selenium produced FeSe_2_, and the one with preformed red selenium still had a significant amount of Fe_7_Se_8_ (judging by the relative intensities of the reflections at 34.3° and 34.9° 2θ).

**Figure 5:**
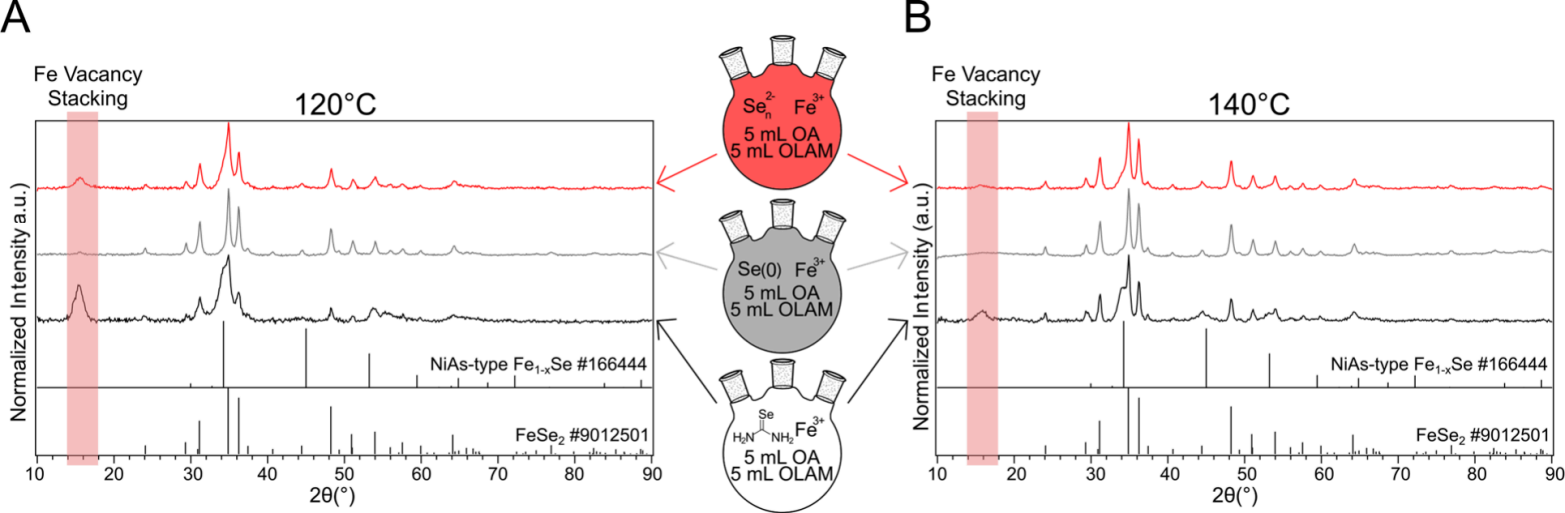
pXRD patterns of synthesized iron selenide nanoparticles using selenourea (bottom, black), gray selenium (middle, gray), or red selenium (top, red) as active selenium precursors at a 50%/50% OA/OLAM mixture and (A) 120 °C and (B) 140 °C.

Finally, in an attempt to further explore the structural differences of red and gray selenium, Raman spectroscopy and light microscopy were performed (Figures S12–S15). Previous reports in literature show that the fingerprint region of Raman contains the most significant information and can be used to differentiate between red and gray selenium allotropes.^43^ Two peaks in the fingerprint region, 240 and 258 cm^–1^, were present for both samples and agree with the literature for selenium allotropes. Due to the low signal-to-noise ratio, no noticeable differences between the two allotropes could be identified, which are known to be low intensity signals below 145 cm^–1^. Light microscopic images of the selenium allotropes show a uniform gray color for the gray selenium sample (Figure S13), whereas the red selenium was reddish pink with other regions that appeared gray, consistent with the XRD study that, in OLAM, the selenourea will decompose into a mixture of red and gray selenium (Figure S15).

## Conclusions

In summary, we successfully synthesized and characterized two of the iron selenide phases using selenourea as a reagent at moderate temperatures of only 100–120 °C. Our research showed that the phases obtained are strongly dependent on the ligand used in the synthesis. While OLAM yielded Fe_7_Se_8_ particles, OA gave rise to FeSe_2_. The ligand influenced which allotrope of the selenium intermediate formed which in turn influenced the iron selenide product phase.

Our understanding of the chemistry happening behind the phase selection is summarized in Figure 6. The phase control resulted from two different decomposition products of selenourea, which acted as intermediates. In the presence of OA, selenourea decomposed into gray Se(0). Byproducts of this reaction included gaseous cyanamide (from the decomposition of selenourea) and carbon dioxide (decarboxylation of OA). Se(0) then reacted with iron­(III) to produce FeSe_2_ nanoparticles. This iron selenide is the most oxidized, where each Se_2_^–2^ unit shares a −2 charge. When reacted with OLAM, selenourea decomposed into red selenium and polyselenides as an intermediate. The gaseous byproduct of this step was ammonia gas. Solubilized ammonium and oleylammonium ions acted as counterions to the polyselenides. This more reduced form of selenium reacted with iron­(III) to form Fe_7_Se_8_, where each selenium carries a −2 charge.

**Figure 6:**
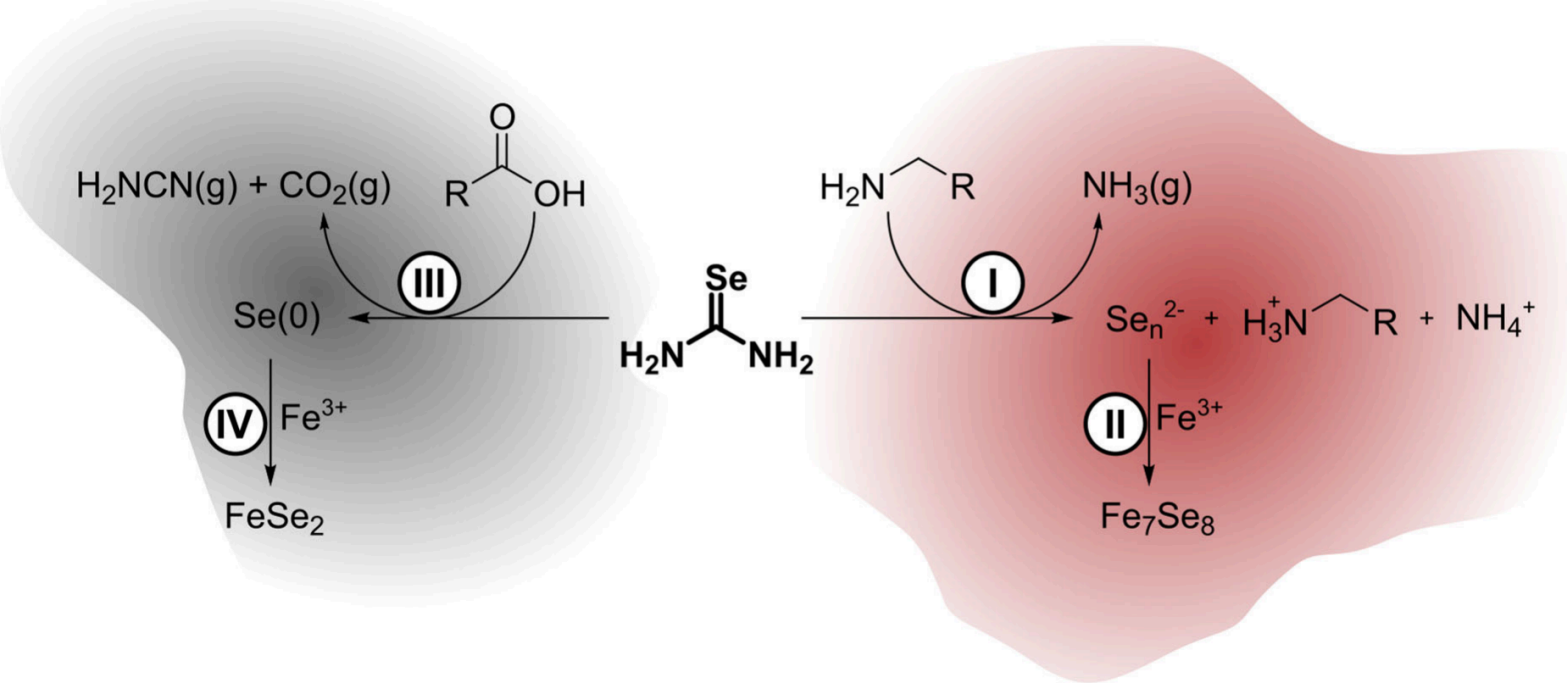
Summary of selenourea decomposition in OLAM and OA: (I) reaction of selenourea and OLAM to produce ammonia gas and red selenium stabilized by oleylammonium and ammonium; (II) reaction of iron and red selenium to produce Fe_7_Se_8_ nanoparticles; (III) reaction of selenourea and OA to produce cyanamide and carbon dioxide gases and gray selenium; (IV) reaction of iron and gray selenium to produce FeSe_2_.

Based on these discoveries, we concluded that the chemistry of selenourea in nanocrystal synthesis did not have a direct correlation to that of thiourea but still showed that ligands influenced the phase that forms. Our previous work had shown that thiourea reacted with OA to form the thiocyanate ion, which is a sulfur source much less reactive than the parent thiourea. The concentration of OA therefore controlled the phase of the iron, cobalt, nickel, and copper sulfides that formed. In all cases, the decomposition of thiourea was catalyzed by the presence of the metal ions. In the synthesis of iron selenides, the decomposition of the selenourea to intermediates of red or gray selenium was independent of the presence of the iron species. These two intermediates, in turn, yielded the two product phases of iron selenides.

We and other groups have used substituted thioureas to great success for size and phase control of metal sulfides.^44–47^ The effect has been ascribed to changing rates of decomposition of the precursor, which influences nucleation and growth. Thiourea has been shown to first coordinate to the metal before decomposition, and therefore, the rate-limiting chemistry for nanocrystal formation is greatly influenced by the substitution pattern of the thiourea. In contrast, here we discovered that selenourea decomposes independently of the metal, and this may be true for substituted selenoureas as well. While the decomposition rate of selenourea will be influenced by the substitution pattern, the process does not occur on the metal, and therefore, it is not guaranteed to be the rate-determining step in the nanocrystal formation. We may not be able to achieve the same level of success in using substituted selenoureas as substituted thioureas for nuanced size and phase control of metal chalcogenides.

Many transition metal selenides have multiple phases with Se_2_^2–^ and Se^2–^ ions, including Mn, Co, Ni, Cu, Pd, Rh, and many others. This research has shown that these ions can be selected for by the solvent choice when using selenourea as a reagent. This understanding will lead to further control of phase of other metal selenides spanning the periodic table.

## Experimental Section

### Materials

Iron­(III) stearate was purchased from the Tokyo Chemical Industry. The following chemicals were purchased from Sigma-Aldrich: selenourea (98%), oleylamine (70%, distilled *in vacuo* and stored in a glovebox), tetraglyme (99%) (sparged with N_2_ for 16 h and stored over molecular sieves), oleic acid (90%, sparged with N_2_ for 16 h and stored in a glovebox), anhydrous diglyme (99.5%, distilled over Na/sodium benzophenone under N_2_ and stored in a glovebox). Deuterated dimethyl sulfoxide (99.9%) was purchased from Cambridge Isotope Laboratories, Inc.

### Synthesis

#### Synthesis of Iron Selenide Nanoparticles by Selenourea Injection (Full Scale)

In a typical nanocrystal synthesis, 0.25 mmol of iron­(III) stearate was loaded in a 25 mL three-neck round-bottom flask and then transferred inside the glovebox along with an empty 5 mL pear shaped flask. Inside the glovebox, 5 mL of OA or OLAM was added to the round-bottomed flask, and 0.75 mmol of selenourea and 5 mL of dry diglyme were added to the pear flask (for 200 °C studies, dry tetraglyme was used instead of diglyme and was added outside of the glovebox). The glassware was then transferred outside of the glovebox, and the round-bottomed flask solution was vacuumed at 100 °C for 30 min. The iron containing solution was heated to 100–200 °C under constant N_2_ flow on the Schlenk line, and the selenourea solution was injected via a syringe. The reaction was allowed to continue for 1 h. Upon cooling, the resulting nanocrystals were precipitated with isopropyl alcohol (15 mL), centrifuged (8000 rpm), and resuspended with hexanes (5 mL) three times.

#### Synthesis of Iron Selenide Nanoparticles (NMR Scale)

The following procedure was adapted from Koziel et al.^48^ An NMR tube was loaded with 0.1 mmol of selenourea, 0.1 mmol of a ligand (OA or OLAM), and/or 0.2 mmol of the solvent (diglyme) in a glovebox and sealed with a septum. Once removed from the glovebox, the NMR tube was placed into a preheated oil bath (120–160 °C) with an N_2_ filled balloon affixed to the top. The reaction was heated for 60 min. Upon cooling, 600 μL of DMSO-*d*_*6*_ was added, and ^1^H and ^13^C NMR spectra were taken.

#### Synthesis of Iron Selenide Nanoparticles by Iron­(III) Stearate Injection (Controls)

0.25 mmol portion of iron­(III) stearate was loaded in a 5 mL pear shaped flask and then transferred inside the glovebox along with an empty 25 mL three-neck round-bottom flask. Inside the glovebox, the 5 mL pear flask was loaded with 5 mL of OA or OLAM and the 25 mL round-bottom flask was loaded with 0.75 mmol of selenourea, 5 mL of dry diglyme, and 2.5 mL of OA or OLAM. Outside the glovebox, the selenium containing solution was heated to 140 °C under constant N_2_ flow on the Schlenk line for 1 h. Then, the iron solution was sonicated and injected into a selenium solution. The reaction was allowed to continue for 1 h. Upon cooling, the resulting nanocrystals were precipitated with isopropyl alcohol (15 mL), centrifuged (8000 rpm), and resuspended with hexanes (5 mL) three times.

#### Synthesis of Red and Gray Selenium (Controls)

In the glovebox, a 25 mL three-neck round-bottom flask was loaded with 5 mL of OA or OLAM, and a 5 mL pear shaped flask was loaded with 0.75 mmol of selenourea and 5 mL of dry diglyme. Outside the glovebox, the OA or OLAM containing solution was placed under vacuum at 100 °C for 30 min. Then, the solution was heated to 140 °C, and selenourea dissolved in diglyme was swiftly injected via a syringe. The reaction was then allowed to continue for 1 h. Upon cooling, the resulting selenium particles were immediately drop-cast onto a zero-background pXRD plate to prevent any further loss of material. The product after synthesis in OLAM was a red powder; in OA, the product was gray.

#### Synthesis of Iron Selenide Nanoparticles with Elemental Selenium at Solvent Mixtures

A 5 mL pear shaped flask was loaded with iron­(III) stearate (0.25 mmol) and moved into the glovebox along with another 5 mL pear shaped flask and a 25 mL three-neck round-bottom flask. For the synthesis with gray selenium, in the glovebox, a 25 mL three-neck round-bottom flask was loaded with 5 mL of OA; the iron-containing 5 mL pear shaped flask was loaded with 5 mL of OLAM; and the empty 5 mL pear shaped flask was loaded with 0.75 mmol of selenourea and 5 mL of dry diglyme. For the synthesis with red selenium, in the glovebox, a 25 mL three-neck round-bottom flask was loaded with 5 mL of OLAM; the iron-containing 5 mL pear shaped flask was loaded with 5 mL of OA; and the empty 5 mL pear shaped flask was loaded with 0.75 mmol of selenourea and 5 mL of dry diglyme. Outside the glovebox, the three-neck round-bottom flask was placed under vacuum at 100 °C for 30 min. Then, the solution was heated to either 120 or 140 °C, and selenourea dissolved in diglyme was swiftly injected via a syringe. The reaction was then allowed to continue for 1 h. Then, the iron-containing solution was injected and allowed to react for an additional 1 h. Upon cooling, the resulting nanocrystals were precipitated with isopropyl alcohol (15 mL), centrifuged (8000 rpm), and resuspended with hexanes (5 mL) three times.

#### Preparation of Samples for Gas FTIR Analysis

For the studies without the metal, a 25 mL three-neck round-bottom flask was loaded with selenourea (0.75 mmol) and OLAM (15.2 mmol) or OA (15.8 mmol) and heated to the specified temperature. For the studies with the metal, a 25 mL three-neck round-bottom flask was loaded with iron­(III) stearate (0.25 mmol) and OLAM (15.2 mmol) or OA (15.8 mmol) and heated to 100 °C. A solution of selenourea (0.75 mmol) and diglyme (34.9 mmol) inside of a 5 mL pear shaped flask was then injected, and the solution was heated to around 160 °C. For the studies without the metal, all reagents were prepared inside of the glovebox and were not degassed prior to FTIR measurements. For the studies with the metal, iron was packed outside the glovebox and then transferred inside of it. Then, the rest of the reagents were prepared inside the glovebox, and outside the glovebox, the three-neck-flask was placed under vacuum at 100 °C for 30 min before refilling with N_2_ prior to FTIR measurements.

For additional diglyme studies done on the OA system, a 25 mL three-neck round-bottom flask was loaded with selenourea (0.75 mmol), OA (15.8 mmol), and diglyme (34.9 mmol) inside the glovebox. No heated degassing was performed for this sample. The flask was transferred to a gas FTIR cell and heated to a specified temperature.

For additional high temperature studies done on the OA system, a 25 mL three-neck round-bottom flask was loaded with iron­(III) stearate (0.25 mmol) and transferred inside of the glovebox. Then, selenourea (0.75 mmol) and OA (15.8 mmol) were loaded into a round-bottom flask inside the glovebox. The flask was then taken out of the glovebox, and tetraglyme (22.7 mmol) stored over molecular sieves outside the glovebox was syringed into the round-bottom flask. No heated degassing was performed for this sample. The flask was transferred to the gas FTIR cell and heated to a specified temperature.

For each setup, two gas adapters were attached to the round-bottom flask to allow for the flow of nitrogen gas into and gases out of the flask. The gas IR cell outlet was attached to a bubbler and was flushed with nitrogen to eliminate any atmospheric gases. Then, the IR cell inlet was attached to the flask via a gas adapter. The whole system was flushed one more time with nitrogen until the spectrum reached a steady state, and then, the nitrogen flow rate was reduced to and kept at ∼2 bubbles per second. Finally, the flask was heated, and the IR spectra of the outflowing gases were collected approximately every 10 °C from 25 to 160 °C when diglyme was present and from 25 to 200 °C for all other experiments.

### Characterization

^1^H and ^13^C NMR spectra were recorded on a 400 MHz Bruker console wuipted with an 11.7 T Oxford Magnet and a 5 mm Z-gradient broad band probe. The samples were diluted with 600 μL of DMSO-*d*_*6*_. All spectra were recorded at 25 °C. Powder X-ray diffraction (pXRD) samples were prepared through drop-casting suspensions of nanocrystals onto the silicon pXRD wafers. pXRD was taken on a Rigaku SmartLab X-ray diffractometer with an operating voltage of 40 kV and a current of 44 mA. pXRD used a Cu Kα source (λ = 0.154 nm) and a D/TeXUltra250 detector (step size of 0.1). All samples were run at 25 °C. Transmission electron microscopy (TEM) images and high-angle annular dark-field scanning transmission electron microscopy coupled with energy dispersive X-ray spectroscopy (HAADF-STEM-EDS) were acquired using an FEI Tecnai Osiris TEM operated at 200 keV. For gas FTIR, a procedure from Shults et al. was followed, but calcium fluoride (CaF_2_) windows were used instead of KRS5 windows.^31^ After the synthesis of red and gray selenium, the powders were added to glass slides and positioned inside the Raman microscope for analysis. Raman spectroscopy and microscopy were performed on a confocal Raman microscope Thermo Scientific DTX. The samples were analyzed with a green laser (532 nm) at 10 mW. Light microscopic images were collected at either 10× or 50× magnification.

## Supplementary Material



## Notes

Powder X-ray diffraction patterns of main syntheses, control experiments, and standards; gas Fourier transform infrared spectra of gas evolution in the presence of diglyme and tetraglyme; Raman spectroscopy and microscopy of gray and red selenium; transmission electron microscopy images of FeSe_2_ and Fe_7_Se_8_; nuclear magnetic resonance spectra of the decomposition products in the presence of oleylamine and oleic acid (PDF)

## Abbreviations

pXRD: powder X-ray diffraction
NMR: nuclear magnetic resonance
FTIR: Fourier transform infrared
OLAM: oleylamine
OA: oleic acid
TEM: transmission electron microscopy
HAADF-STEM-EDS: high-angle annular dark-field scanning transmission electron microscopy coupled with energy dispersive X-ray spectroscopy
