# Supplementary material for: Decomposition of Selenourea in Various Solvents: Red versus Gray Selenium in the Synthesis of Iron Selenide Nanoparticles
Source: Chem Mater. 2025 Jun 10;37(12):4282–90. doi: 10.1021/acs.chemmater.4c03430 (PMC12199294; doi:10.1021/acs.chemmater.4c03430)
Supplement: Supplementary file 1 [file cm4c03430_si_001.pdf]

Decomposition of Selenourea in Various Solvents: Red versus Gray Selenium in the Synthesis of Iron Selenide Nanoparticles

Andrey A. Shults[1,2] *Alexandra C. Koziel,*[1,2] *Joshua D. Caldwell,*[1,2,3] *and Janet E. Macdonald**[1,2]

[1]Department of Chemistry, Vanderbilt University, Nashville TN 37235, USA

[2]Vanderbilt Institute for Nanoscale Science and Engineering, Nashville TN 37235, USA

[3]Department of Mechanical Engineering, Vanderbilt University, Nashville TN 37235, USA

*janet.macdonald@vanderbilt.edu

Contents

1. Powder X-Ray Diffraction (pXRD) Patterns 4

1.1 Raw pXRD spectra for every solvent ratio and reaction temperature 4

1.2 Iron Injection into Selenourea Decomposed in Oleylamine 5

1.3 Iron Injection into Selenourea Decomposed in Oleic Acid 6

1.4 Synthesis of Red Selenium in the Presence of Oleylamine 7

1.5 pXRD Standards of Selenourea, Iron (III) Stearate, and an Oleylamine/Oleic Acid Mixture 8

1.6 pXRD Patterns of the Aliquot Studies Performed on the Reaction at 100% OLAM and 140°C 9

2. Gas Fourier Transform Infrared (FTIR) Spectra 10

2.1 Full Gas Phase FTIR Spectrum of the OLAM System 10

2.2 Full Gas Phase FTIR Spectrum of the OA System 10

2.3 Gas Evolution of Selenourea in the presence of Diglyme and Oleic Acid 11

2.4 High Temperature Gas Evolution in the Presence of Tetraglyme 12

2.5 Temperature Studies of the Reaction Between Selenourea and Oleylamine 13

3. Raman Spectroscopy and Microscopy of Gray and Red Selenium 14

3.1 Raman Spectrum of Gray Selenium 14

3.2 Microscopic Image of Gray Selenium 14

3.3 Raman Spectrum of Red Selenium 15

3.4 Microscopic Image of Red Selenium 15

4. Transmission Electron Microscopy Coupled with High-Angle Annular Dark-Field Scanning Transmission Electron Microscopy and Energy-Dispersive X-ray Spectroscopy (TEM-HAADF-STEM-EDS) 16

4.1 Microscopic Images of $FeSe_2$ Nanoparticles Synthesized in the Presence of Oleic Acid for 10 minutes 16

4.2 Microscopic Images of $FeSe_2$ Nanoparticles Synthesized in the Presence of Oleic Acid for 60 Minutes 17

4.3 Microscopic Images of Fe_7Se_8 Nanoparticles Synthesized in the Presence of Oleylamine for 60 Minutes 18

5. Nuclear Magentic Resonance (NMR) Spectra 19

5.1 ^{13}C NMR Temperature Studies of Oleylamine Syntheses 19

5.2 ^{13}C NMR Temperature Studies of Oleylamine Syntheses Zoomed In 20

5.3 ^{13}C NMR Temperature Studies of Oleic Acid Syntheses 21

5.4 ^{13}C NMR Temperature Studies of Oleic Acid Syntheses Zoomed In 22

5.5 ^{1}H NMR Temperature Studies of Oleylamine and Diglyme Syntheses 23

5.6 ^{13}C NMR Temperature Studies of Oleylamine and Diglyme Syntheses 24

5.7 ^{13}C NMR Temperature Studies of Oleylamine and Diglyme Syntheses Zoomed In 25
5.8 ^{1}H NMR Temperature Studies of Oleic Acid and Diglyme Syntheses 26
5.9 ^{13}C NMR Temperature Studies of Oleic Acid and Diglyme Syntheses 27
5.10 ^{13}C NMR Temperature Studies of Oleic Acid and Diglyme Syntheses Zoomed In 28
6. References...29

1. Powder X-Ray Diffraction (pXRD) Patterns

1.1 Raw pXRD spectra for every solvent ratio and reaction temperature

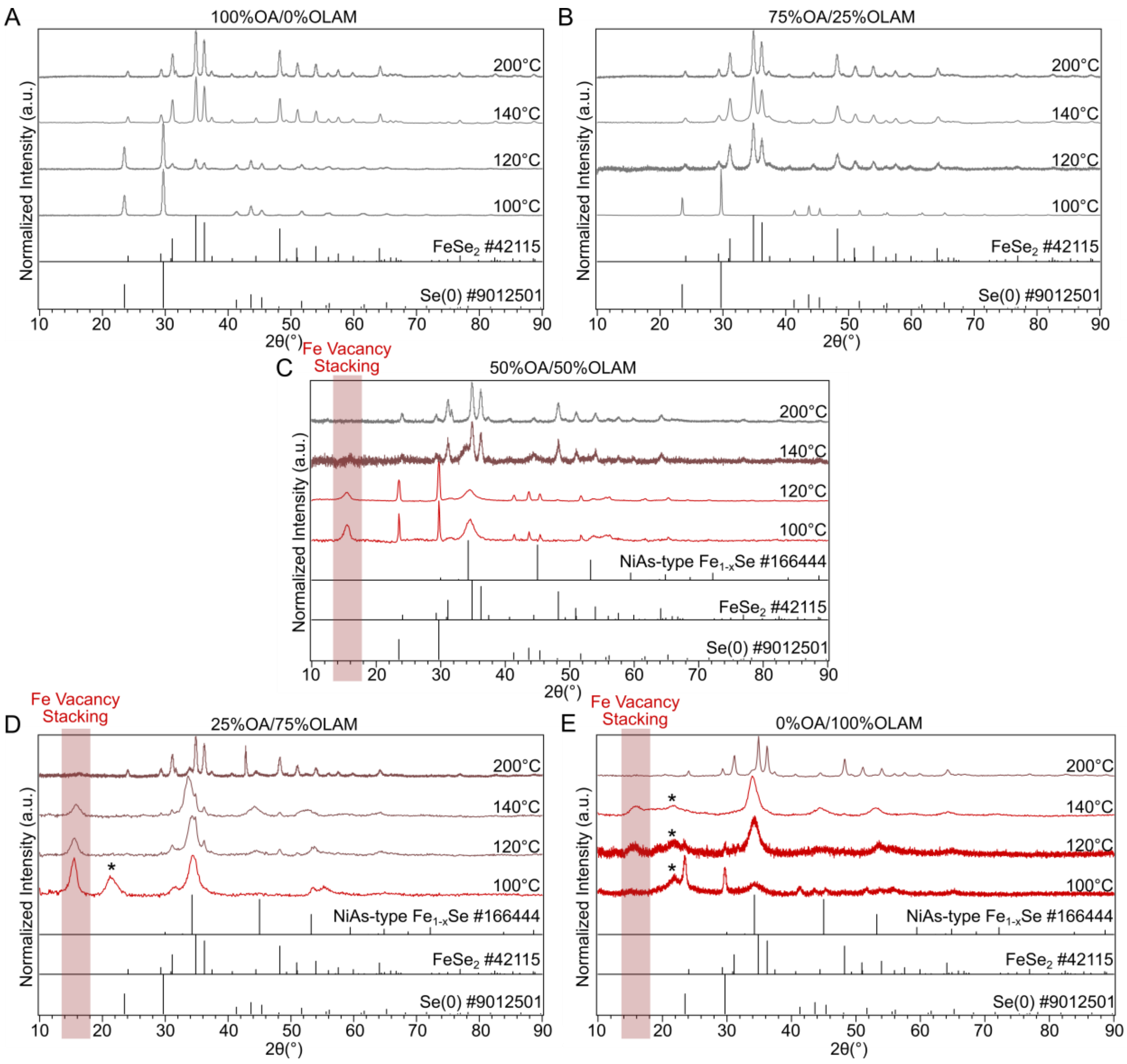

Figure S1. pXRD patterns of nanoparticle phases resulting from the reaction of iron(III) stearate dissolved in 5 mL of OA and/or OLAM and selenourea dissolved in 5 mL of diglyme or tetraglyme (200ºC) for 1h. A) 100%OA:0%OLAM. B) 75%OA:25%OLAM. C) 50%OA:50%OLAM D) 25%OA:75%OLAM. E) 0%OA:100%OLAM. * indicate a peak that correlates to the unreacted iron(III) stearate precursor.

1.2 Iron Injection into Selenourea Decomposed in Oleylamine

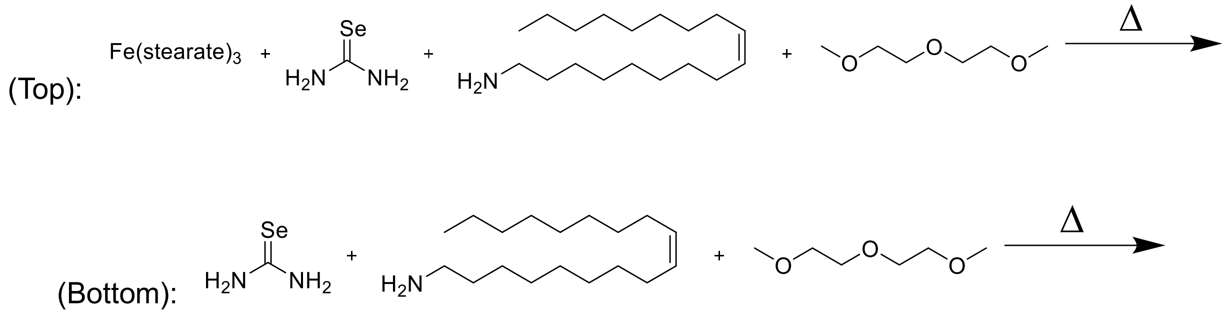

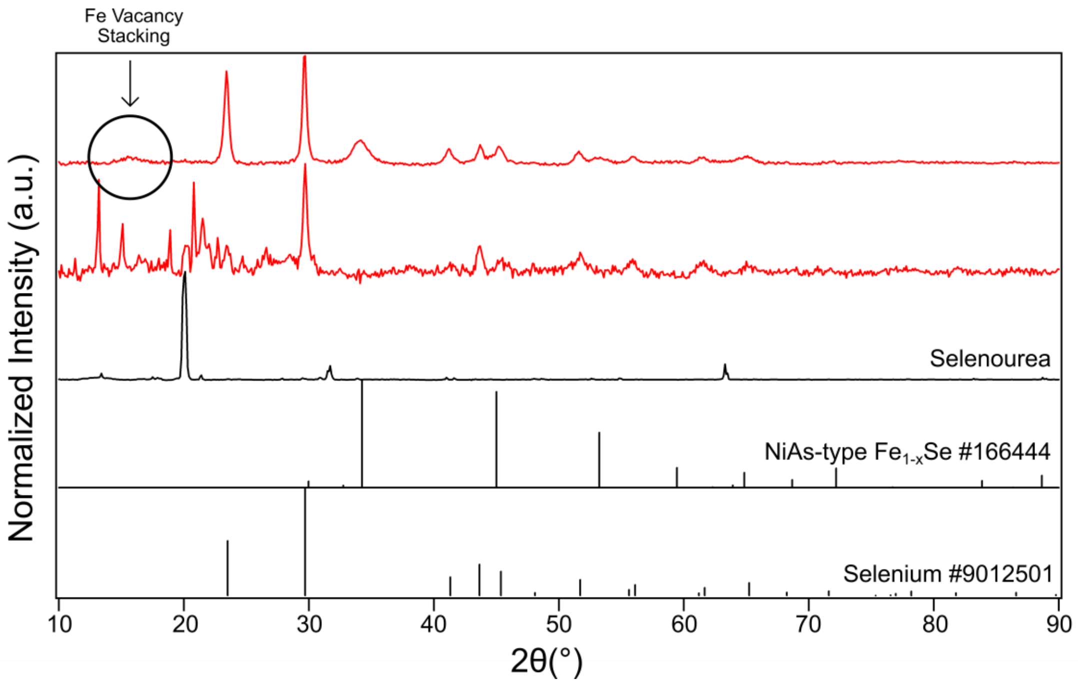

Figure S2. pXRD control studies of the oleylamine system: reaction between selenourea, oleylamine, and diglyme at 140 ºC for 1 hr (bottom) and reaction between iron (III) stearate, selenourea, oleylamine, and diglyme where iron was injected into selenium after reaction selenourea, oleylamine, and diglyme for an hour at 140 ºC (top). Arrow in the top pattern points to the reflection responsible for the stacking of the Fe vacancies. The stacking faults are an indicator of the formation of Fe_7Se_8.

Discussion of the pXRD Control Studies of the Oleylamine System

The first control study (bottom) shows that after reacting selenourea with oleylamine and diglyme at 140 °C, the major products are Se(0) and a molecular species (due to the low angle reflections). The reflections of the molecular species do not match the reflections for selenourea **(Figure S5)**. While there are some similar features of the pattern to the three known polymorphs of Se_8 red selenium, they do not match exactly. The known polymorphs include α (ICSD#9011116),[1] β (ICSD#2204812),[2] and γ (ICSD#9012104),[3] but it is possible the molecular species is an unidentified polymorph of Se_8 or an ammonium polyselenide salt. In the second control study (top), iron (III) stearate was injected into the mixture of selenourea, diglyme, and oleylamine that was preheated for an hour at 140 °C. The reaction was then held for another hour. The resulting nanoparticles are Fe_7Se_8, the same phase as when selenourea is injected into an iron solution. The decomposition of selenourea therefore precedes interaction with iron. Since the Fe_7Se_8 is contaminated with a large amount of Se(0) and the molecular species is missing, it is likely the active source of selenium for nucleating Fe_7Se_8 in the presence of oleylamine is a molecular or ionic polyselenide.

1.3 Iron Injection into Selenourea Decomposed in Oleic Acid

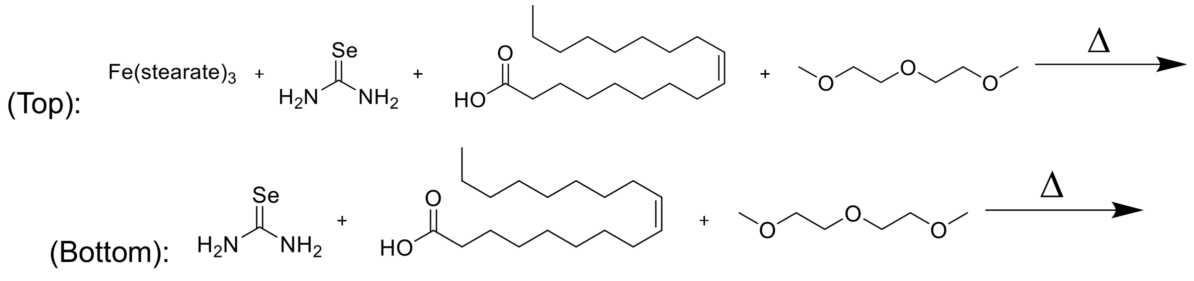

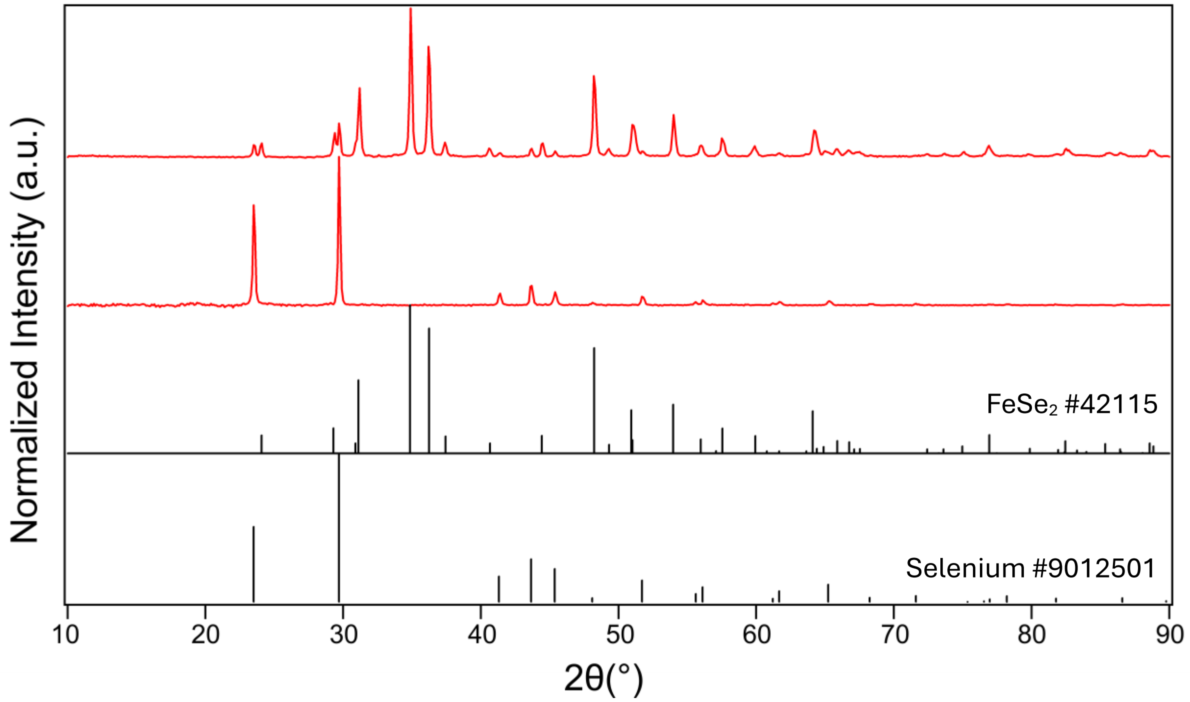

Figure S3. pXRD control studies of the oleic acid system: reaction between selenourea, oleic acid, and diglyme at 140 ºC for 1 hr (bottom) and reaction between iron (III) stearate, selenourea, oleic acid, and diglyme where iron was injected into selenium after reaction selenourea, oleic acid, and diglyme for an hour at 140 ºC (top).

Discussion of the pXRD Control Studies of the Oleic Acid System

The first control study (bottom) shows that after reacting selenourea with oleic acid and diglyme at 140 °C, the major product is gray Se(0). In the second control study (top), iron(III) stearate was injected into the mixture of selenourea, diglyme, and oleic acid that was preheated for an hour at 140°C. The reaction was then held for another hour. The resulting nanoparticles are $FeSe_2$, the same phase as when selenourea is injected into a hot iron solution. The decomposition of selenourea precedes interaction with iron. The active source of selenium for the formation of $FeSe_2$ in the presence of oleic acid is gray selenium.

1.4 Synthesis of Red Selenium in the Presence of Oleylamine

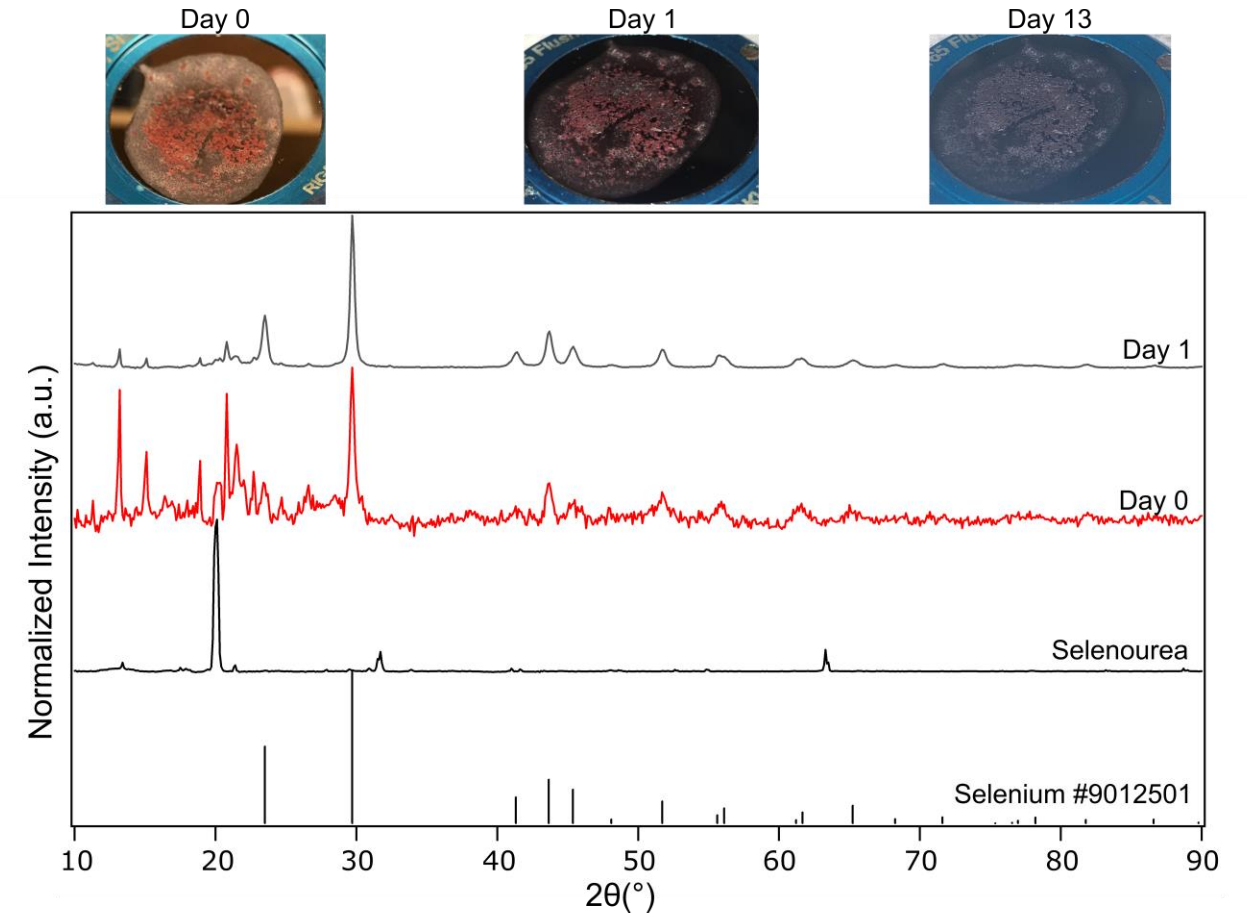

Figure S4. Reaction of oleylamine, diglyme, and selenourea to produce red selenium that later transforms into gray selenium.

1.5 pXRD Standards of Selenourea, Iron (III) Stearate, and an Oleylamine/Oleic Acid Mixture

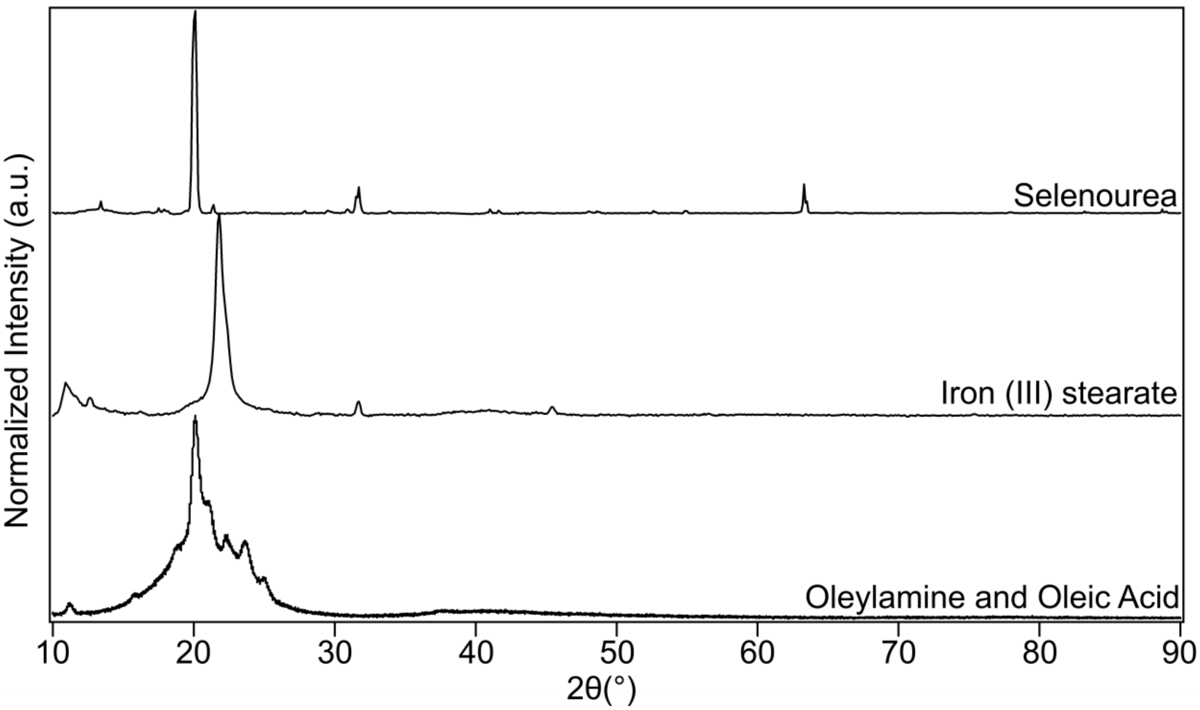

Figure S5. pXRD patterns of precursor standards. From bottom to top: mixture of oleylamine and oleic acid, iron(III) stearate, and selenourea.

1.6 pXRD Patterns of the Aliquot Studies Performed on the Reaction at 100% OLAM and 140 °C

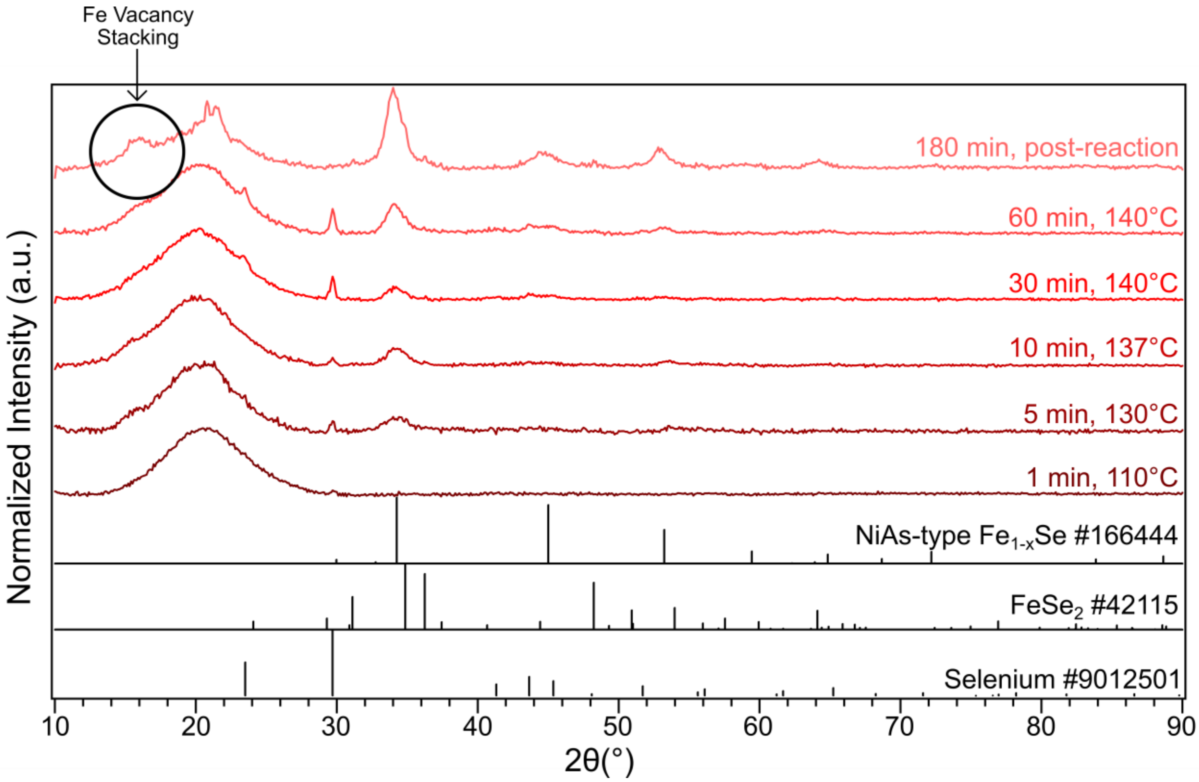

Figure S6. pXRD patterns of the aliquot studies performed on the reaction at 100% OLAM and 140 °C. Aliquots were pulled after the selenourea injection at 1, 5, 10, 30, and 60 minutes. The reaction was stopped at 180 minutes.

2. Gas Fourier Transform Infrared (FTIR) Spectra

2.1 Full Gas Phase FTIR Spectrum of the OLAM System

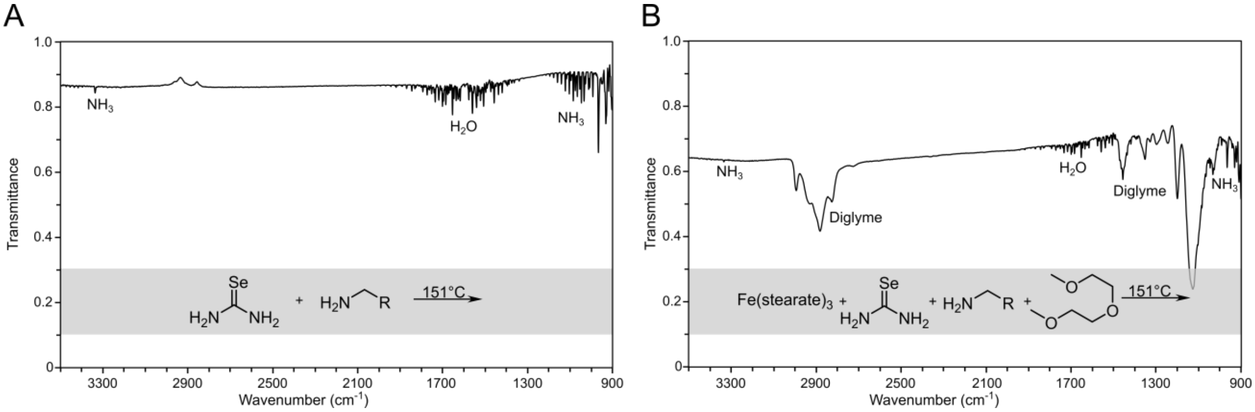

Figure S7. Full gas phase FTIR spectrum of the thermal decomposition products of A) selenourea (0.75 mmol) in oleylamine (15.2 mmol) at 151°C and B) iron(III) stearate (0.25 mmol) and selenourea (0.75 mmol) in oleylamine (15.2 mmol) and diglyme (34.9 mmol) at 151°C

2.2 Full Gas Phase FTIR Spectrum of the OA System

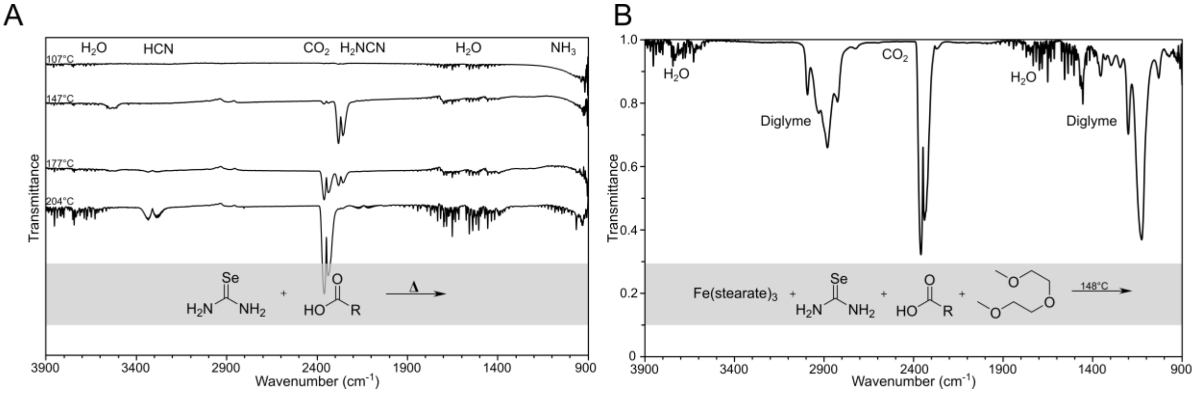

Figure S8. Full gas phase FTIR spectrum of thermal decomposition products of A) selenourea (0.75 mmol) in oleic acid (15.8 mmol) between 107°C and 204°C and B) iron(III) stearate (0.25 mmol) and selenourea (0.75 mmol) in oleic acid (15.8 mmol) and diglyme (34.9 mmol) at 148°C

2.3 Gas Evolution of Selenourea in the presence of Diglyme and Oleic Acid

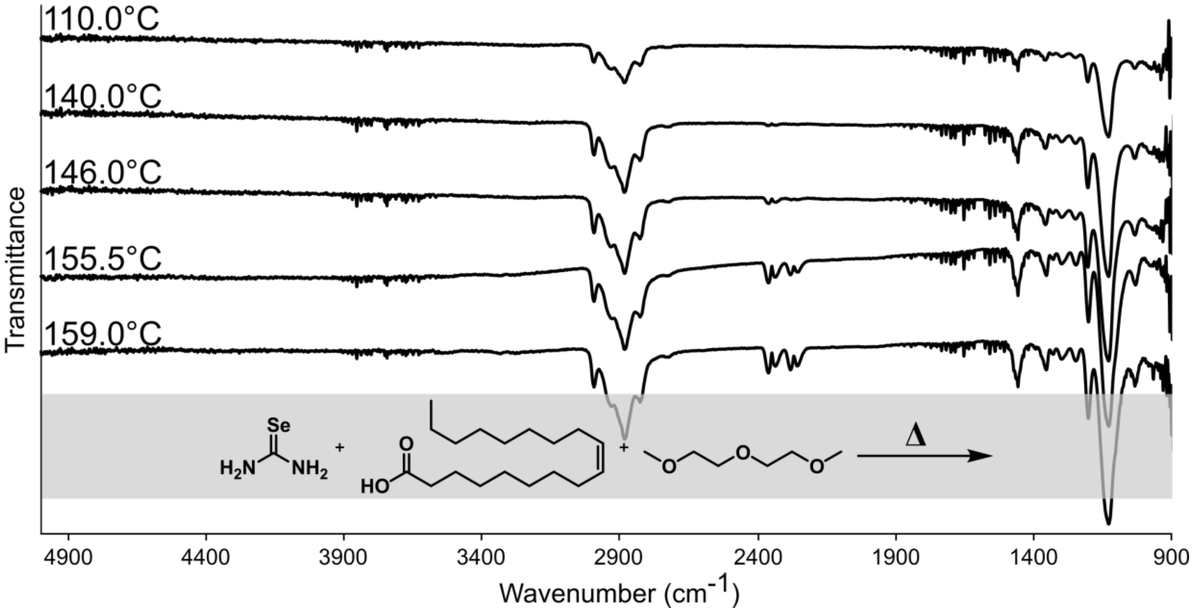

Figure S9. Gas FTIR of thermal decomposition products of selenourea (0.75 mmol) in oleic acid (15.8 mmol) and diglyme (34.9 mmol) at a range of temperatures from 110.0°C to 159.0°C. Peaks at ~2900 cm^{-1} and at ~1300 cm^{-1} are attributed to diglyme. The pair of peaks at 2269 and 2281 cm^{-1} are cyanamide. Peaks at 2340 and 2360 cm^{-1} are carbon dioxide.

Discussion of Gas FTIR Spectra of the Reaction between Selenourea, Diglyme, and Oleic Acid

Presence of diglyme does not stop the evolution of cyanamide. The analysis was stopped at 159°C, as the boiling point of diglyme was reached at 162°C.

2.4 High Temperature Gas Evolution in the Presence of Tetraglyme

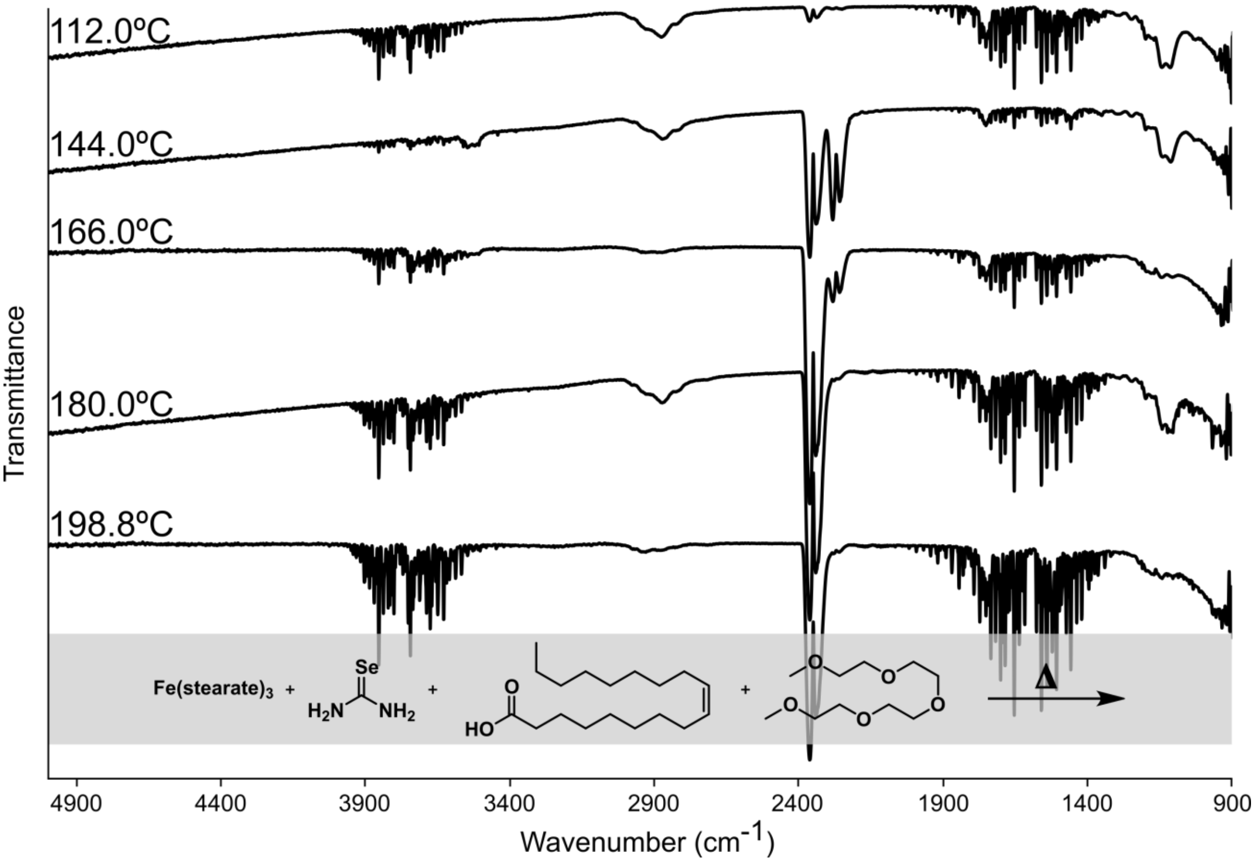

Figure S10. Gas FTIR spectra of the thermal decomposition products of iron (III) stearate (0.25 mmol), selenourea (0.75 mmol) in oleic acid (15.8 mmol) and tetraglyme (22.7 mmol) at a range of temperatures from 110.0°C to 159.0°C. Peaks at ~2900 cm^{-1} are attributed to tetraglyme. The pair of peaks at 2269 and 2281 cm^{-1} are cyanamide. Peaks at 2340 and 2360 cm^{-1} are carbon dioxide.

Discussion of Gas FTIR Spectra of the Reaction between Iron (III) Stearate, Selenourea, Tetraglyme, and Oleic Acid

The cyanamide signal appears at 144 and 166°C yet disappears at higher temperatures. Without the presence of iron **(Figure 3B)**, the loss of the cyanamide was accompanied by the formation of HCN and NH_3. These decomposition products of cyanamide were not observed here. Instead, we ascribe the loss of the cyanamide peak in the gas phase IR to coordination to the iron centerers.

2.5 Temperature Studies of the Reaction Between Selenourea and Oleylamine

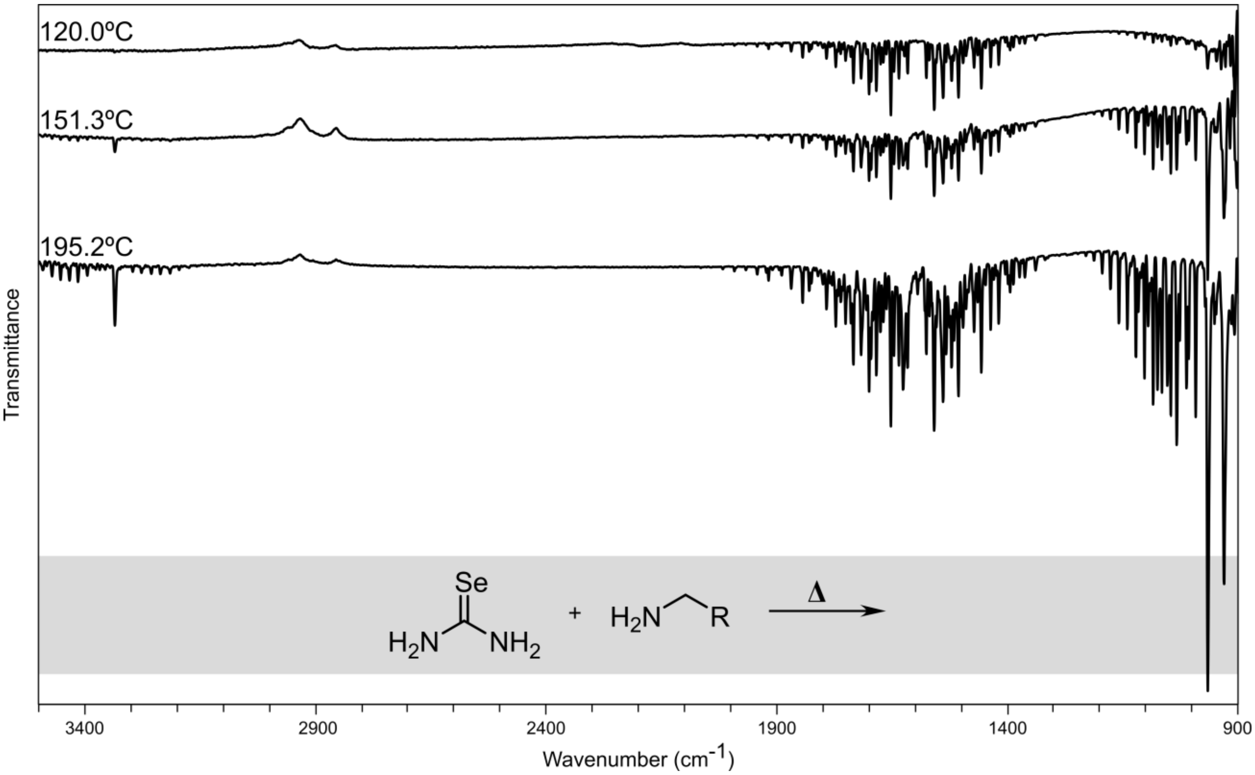

Figure S11. Gas FTIR spectra showing amount of ammonia gas increasing as the reaction temperature increases during the reaction between selenourea (0.75 mmol) and oleylamine (15.2 mmol).

3. Raman Spectroscopy and Microscopy of Gray and Red Selenium

3.1 Raman Spectrum of Gray Selenium

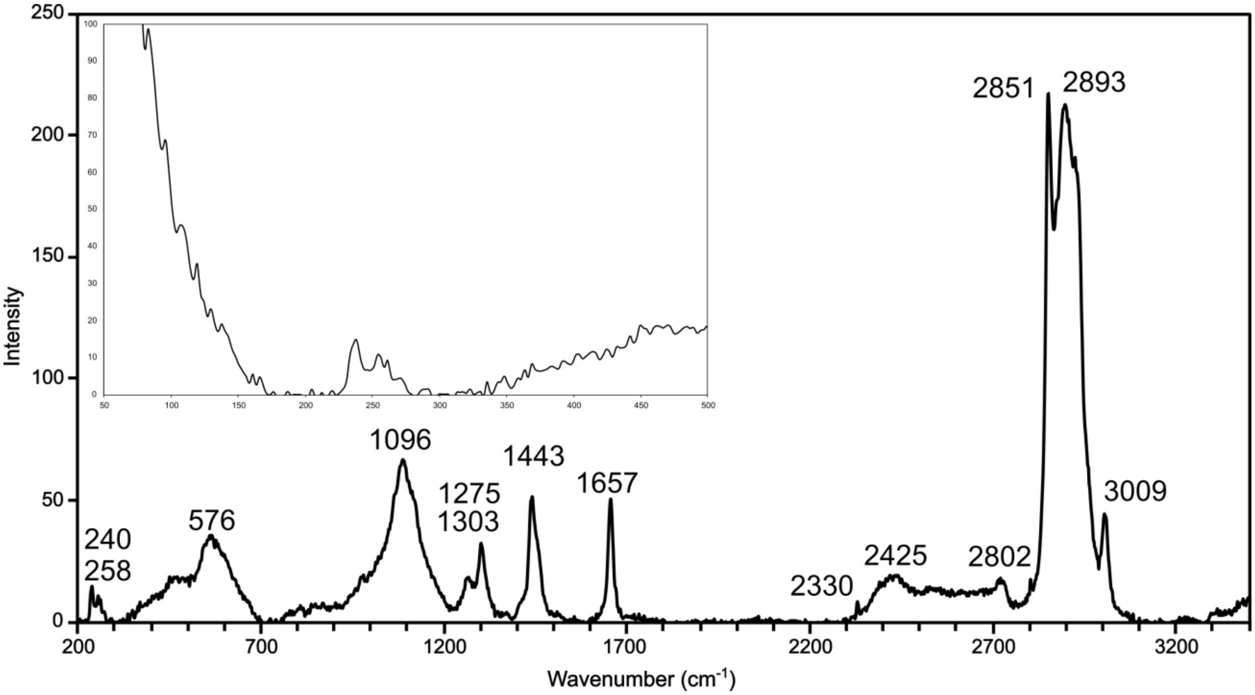

Figure S12. Raman spectrum of gray selenium. The inset in the top left corner shows the fingerprint region of the Raman spectrum. The peaks at 1275, 1303, 1443, and 1657 cm^{-1} that were unique to the gray selenium match well with oleic acid, which was used for the synthesis.[4]

3.2 Microscopic Image of Gray Selenium

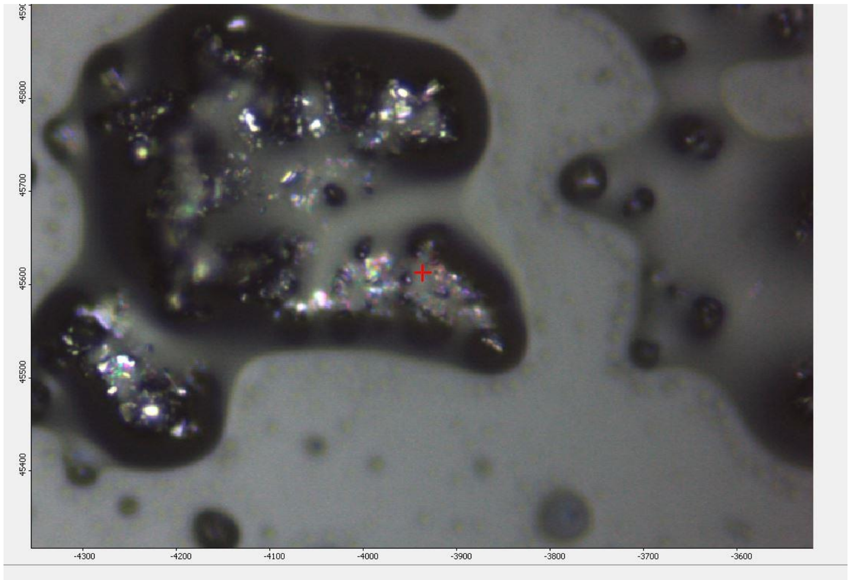

Figure S13. Microscopic image of the gray selenium sample used for Raman spectroscopy.

3.3 Raman Spectrum of Red Selenium

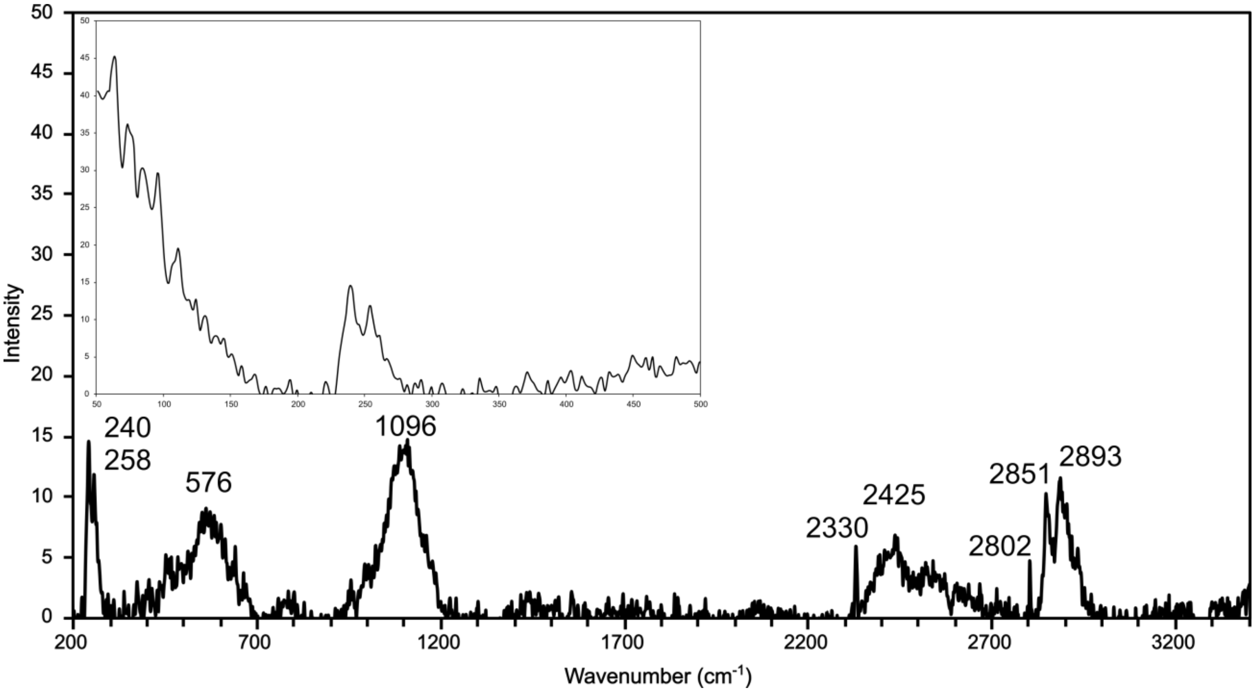

Figure S14. Raman spectrum of red selenium. The inset in the top left corner shows the fingerprint region of the Raman spectrum.

3.4 Microscopic Image of Red Selenium

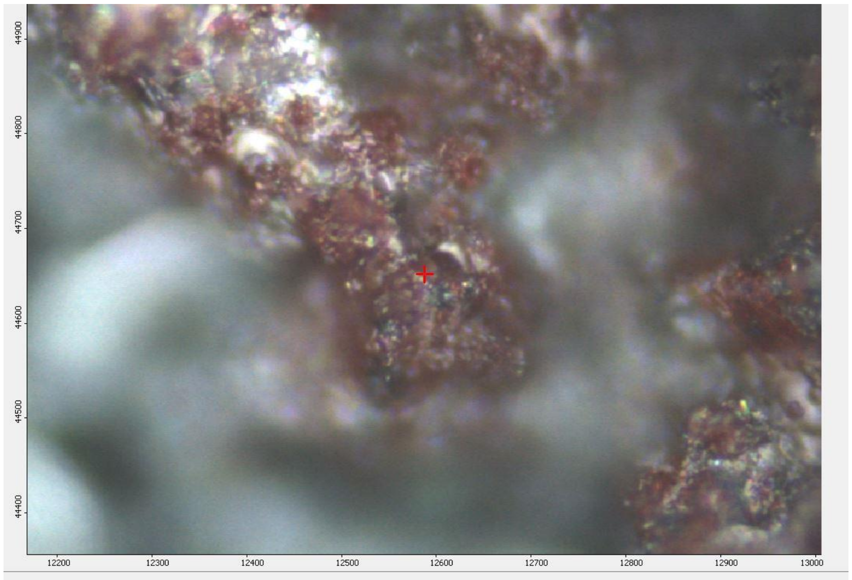

Figure S15. Microscopic image of the red selenium sample used for Raman spectroscopy. Sample shows frequent gray regions resulting from the conversion of red selenium to gray selenium.

4. Transmission Electron Microscopy Coupled with High-Angle Annular Dark-Field Scanning Transmission Electron Microscopy and Energy-Dispersive X-ray Spectroscopy (TEM-HAADF-STEM-EDS)

4.1 Microscopic Images of $FeSe_2$ Nanoparticles Synthesized in the Presence of Oleic Acid for 10 minutes

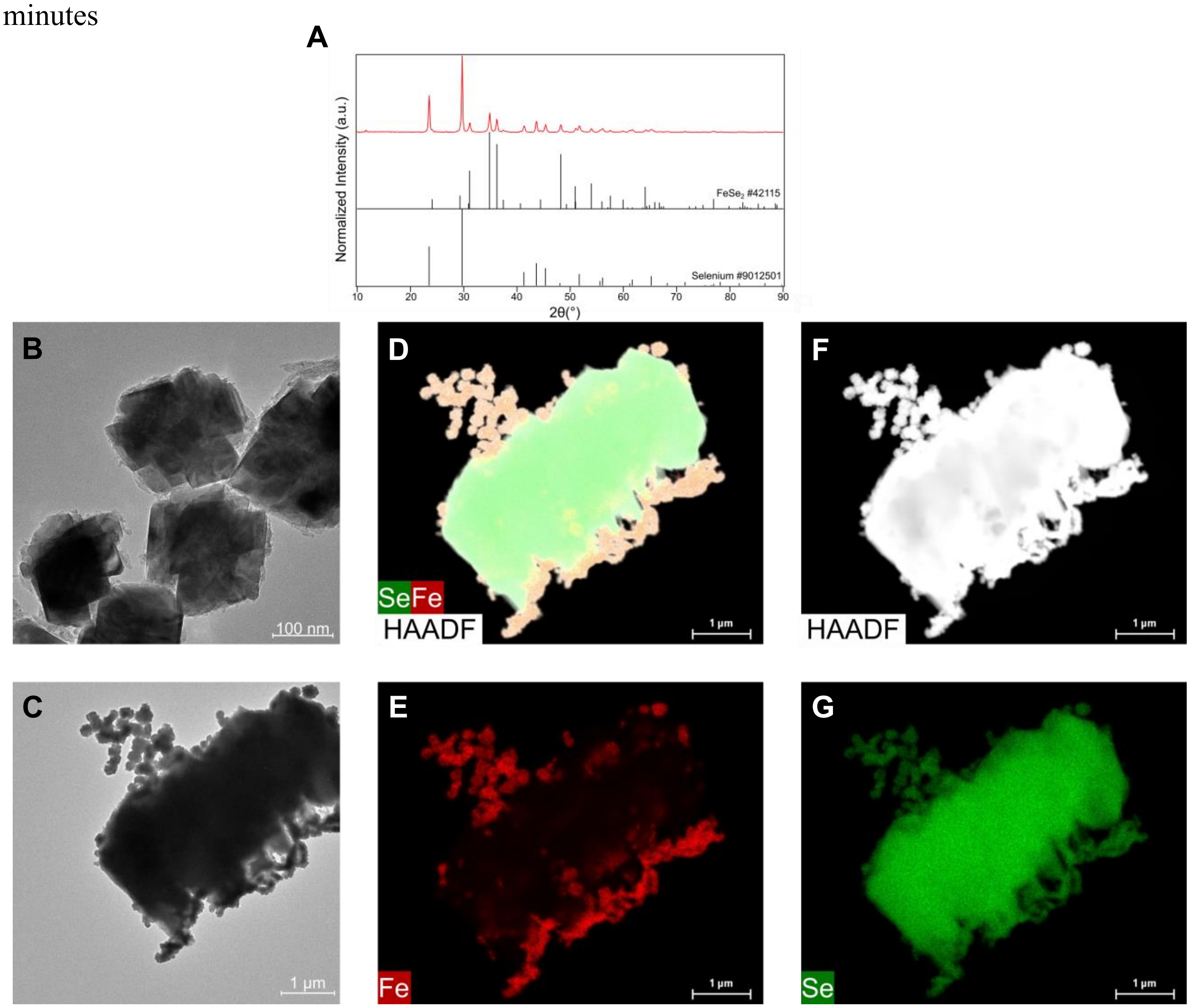

Figure S16. (A) pXRD pattern showing a mixture of $FeSe_2$ and Se(0) synthesized in 100% oleic acid at 10 minutes. Microscopic images of $FeSe_2$ and Se(0) synthesized in oleic acid at 10 minutes including: (B) zoomed in image of iron selenide nanoparticles, (C) combined image of $FeSe_2$ nanoparticles and a selenium particle, (D) combined HAADF, selenium, and iron STEM-EDS, (E) iron STEM-EDS, (F) HAADF of iron selenide nanoparticles, and (G) selenium STEM-EDS.

4.2 Microscopic Images of $FeSe_2$ Nanoparticles Synthesized in the Presence of Oleic Acid for 60 Minutes

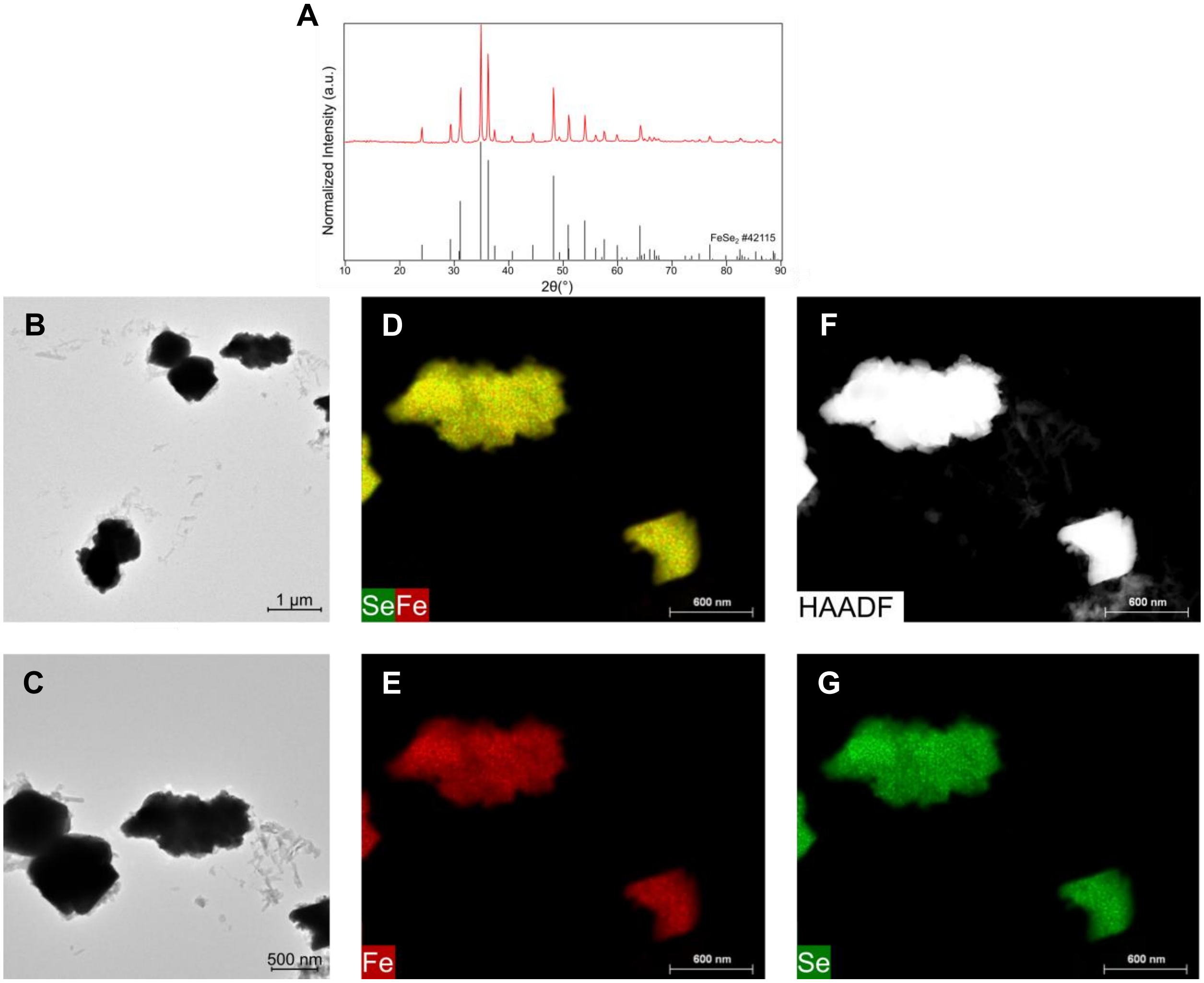

Figure S17. (A) pXRD pattern showing $FeSe_2$ synthesized in 100% oleic acid at 60 minutes. Microscopic image of $FeSe_2$ nanoparticles synthesized in 100% oleic acid at 60 minutes including: (B) zoomed out image of the particle clusters, (C) zoomed in image of the particle clusters, (D) combined selenium and iron STEM-EDS, (E) iron STEM-EDS, (F) HAADF of iron selenide nanoparticles, and (G) selenium STEM-EDS.

4.3 Microscopic Images of Fe_7Se_8 Nanoparticles Synthesized in the Presence of Oleylamine for 60 Minutes

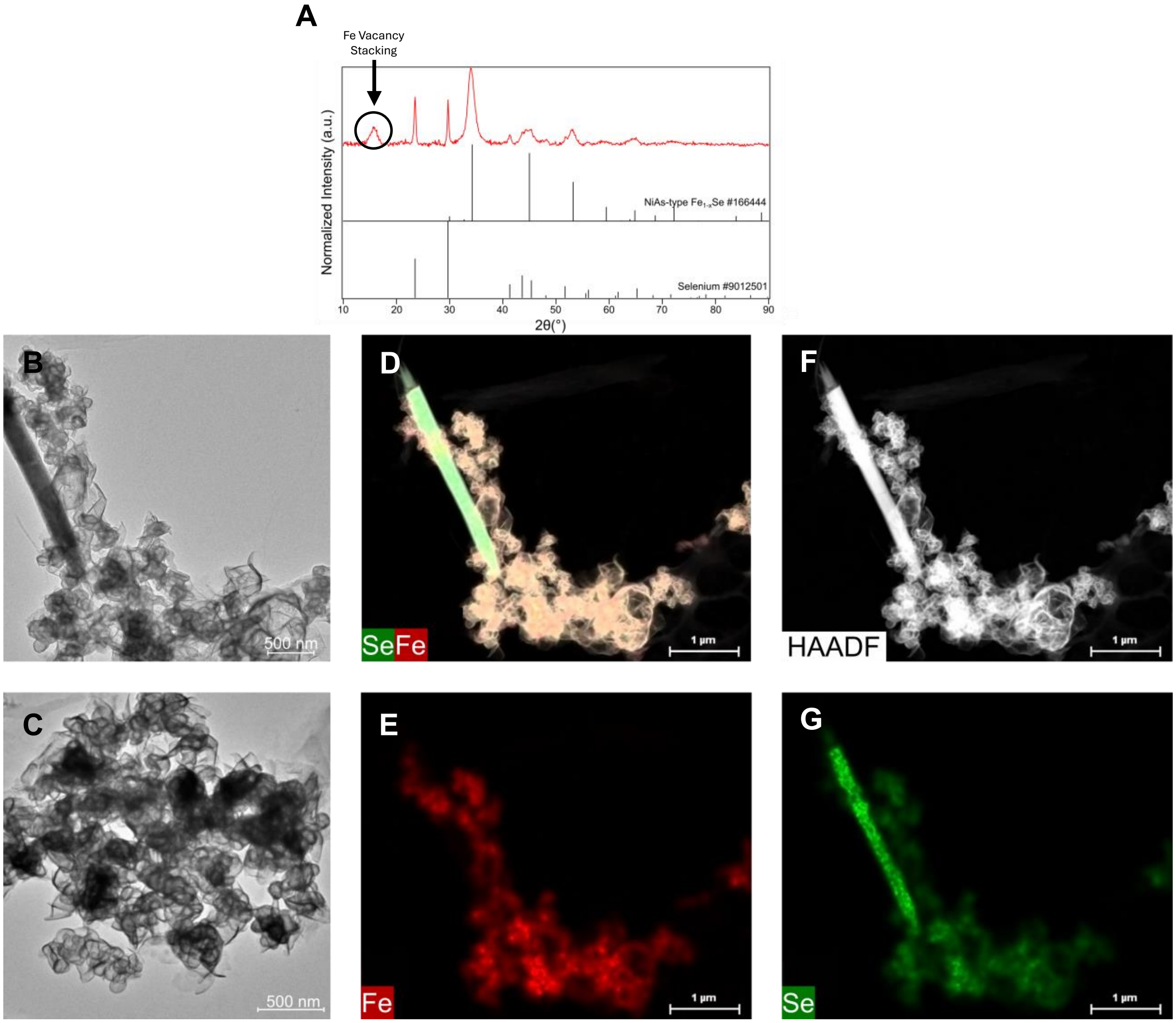

Figure S18. (A) pXRD pattern showing a mixture of Fe_7Se_8 and Se(0) synthesized in 100% oleylamine at 60 minutes. Microscopic images of Fe_7Se_8 and Se(0) synthesized in 100% oleylamine at 60 minutes including: (B) combined image of iron selenide nanoparticles and selenium particles, (C) image of the iron selenide crumbled sheets, (D) combined selenium and iron STEM-EDS, (E) iron STEM-EDS, (F) HAADF of iron selenide nanoparticles, and (G) selenium STEM-EDS.

5. Nuclear Magentic Resonance (NMR) Spectra

5.1 ^{13}C NMR Temperature Studies of Oleylamine Syntheses

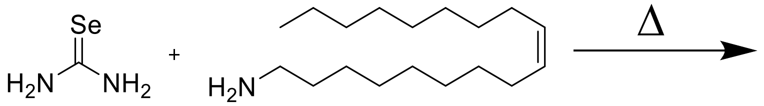

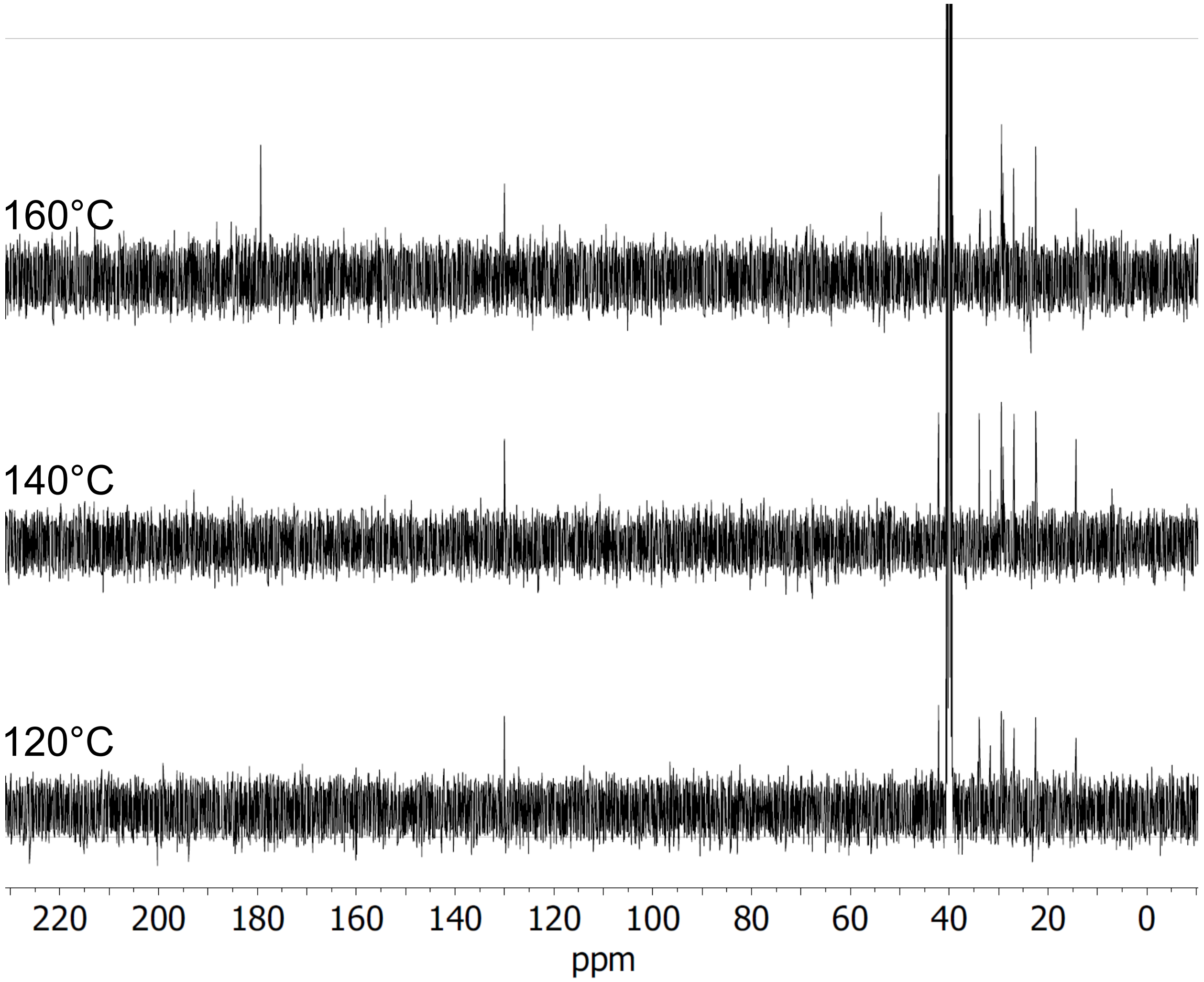

Figure S19. ^{13}C NMR of temperature studies of the reaction between selenourea (0.1 mmol) and oleylamine (0.1 mmol). NMR was performed in 600 μL DMSO-d_6.

5.2 ^{13}C NMR Temperature Studies of Oleylamine Syntheses Zoomed In

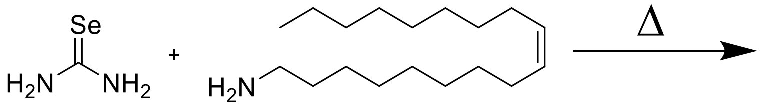

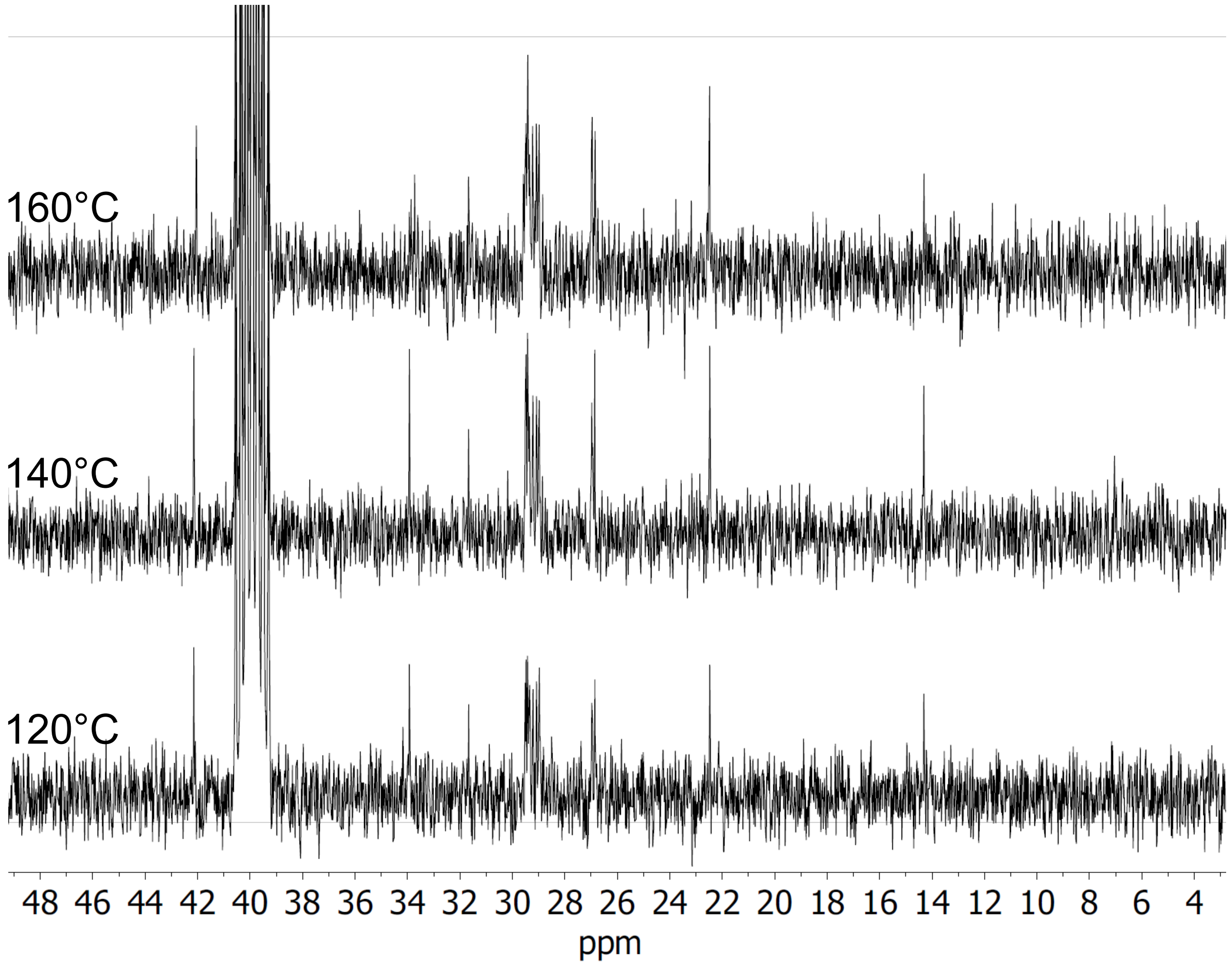

Figure S20. Zoomed in ^{13}C NMR of temperature studies of the reaction between selenourea (0.1 mmol) and oleylamine (0.1 mmol). NMR was performed in 600 μL DMSO-d_6.

5.3 ^{13}C NMR Temperature Studies of Oleic Acid Syntheses

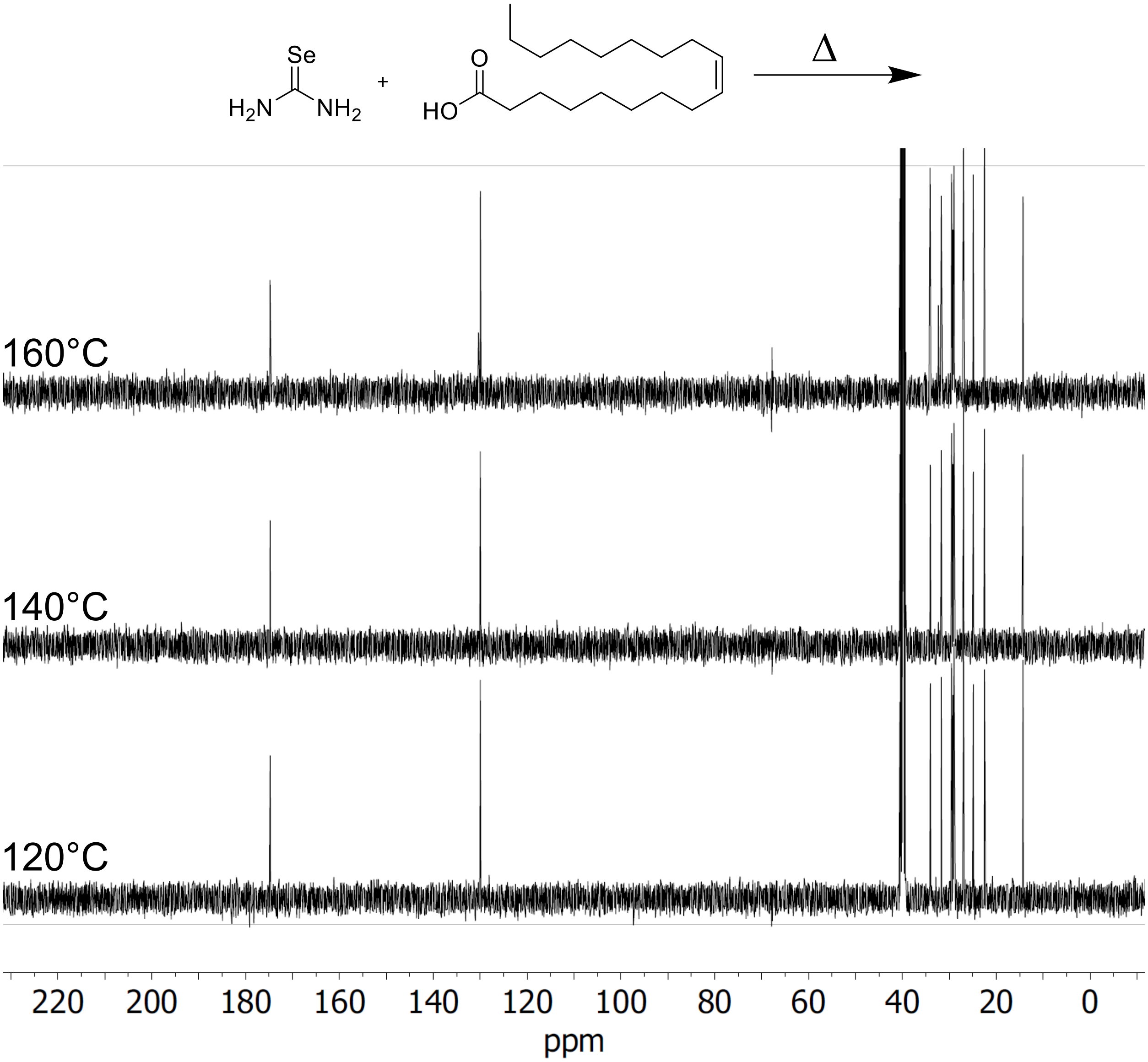

Figure S21. ^{13}C NMR of temperature studies of the reaction between selenourea (0.1 mmol) and oleic acid (0.1 mmol). NMR was performed in 600 μL DMSO-d_6.

5.4 ^{13}C NMR Temperature Studies of Oleic Acid Syntheses Zoomed In

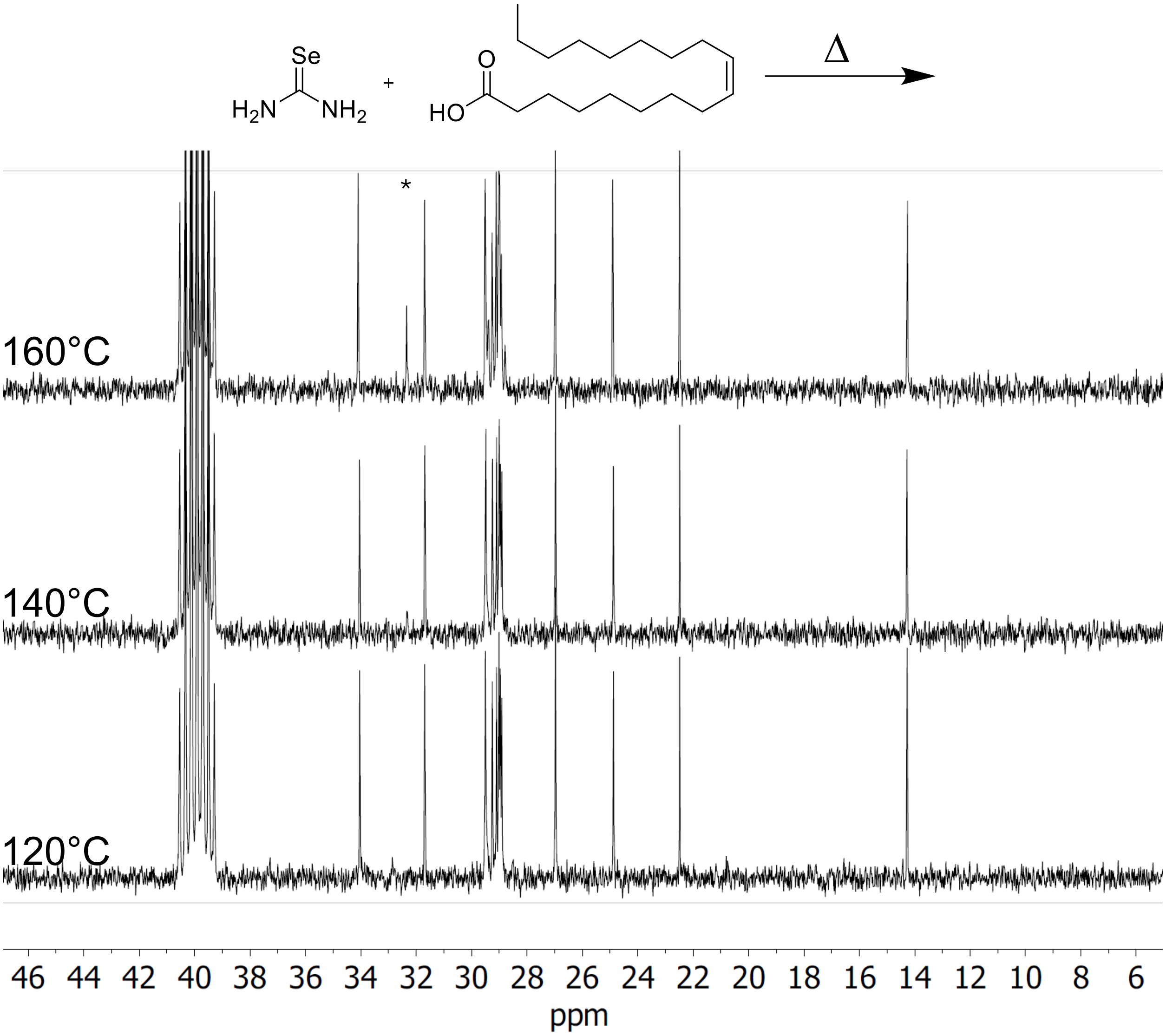

Figure S22. Zoomed in ^{13}C NMR of temperature studies of the reaction between selenourea (0.1 mmol) and oleic acid (0.1 mmol). NMR was performed in 600 μL DMSO-d_6. *We attribute the formation of the peak at around δ = 32 ppm to the cis/trans isomerism of oleic acid into elaidic acid.[5]

5.5 ^{1}H NMR Temperature Studies of Oleylamine and Diglyme Syntheses

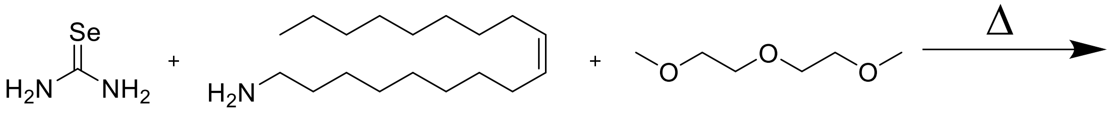

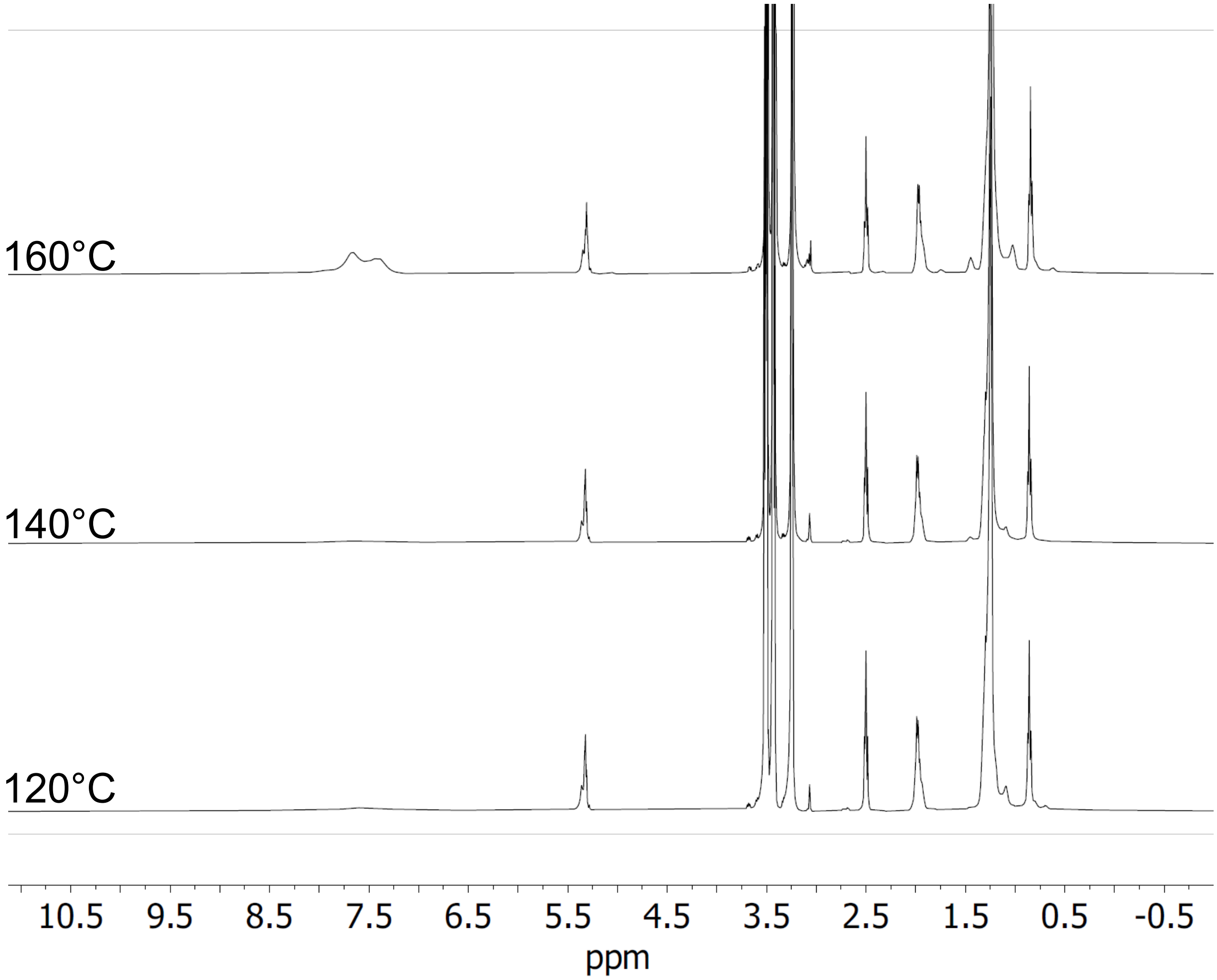

Figure S23. ^{1}H NMR of temperature studies of the reaction between selenourea (0.1 mmol) and oleylamine (0.1 mmol), and diglyme (0.2 mmol). NMR was performed in 600 μL DMSO-d_6.

5.6 ^{13}C NMR Temperature Studies of Oleylamine and Diglyme Syntheses

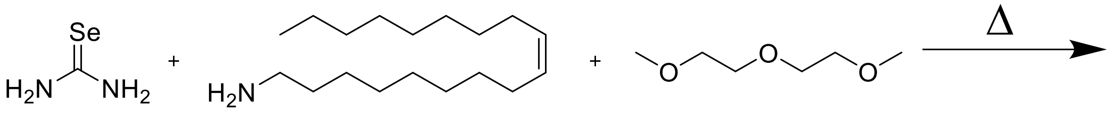

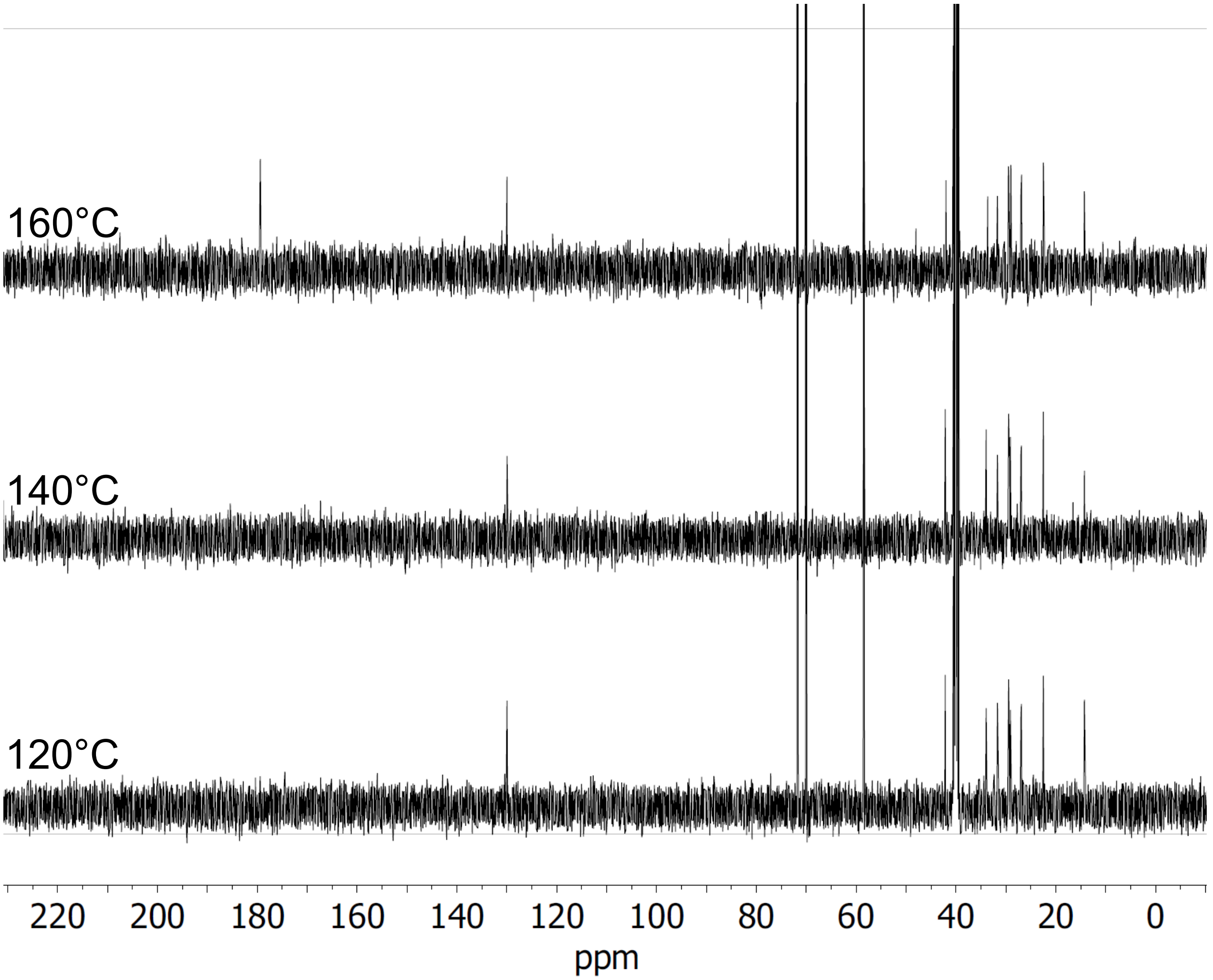

Figure S24. ^{13}C NMR of temperature studies of the reaction between selenourea (0.1 mmol) and oleylamine (0.1 mmol), and diglyme (0.2 mmol). NMR was performed in 600 µL DMSO-d_6.

5.7 ^{13}C NMR Temperature Studies of Oleylamine and Diglyme Syntheses Zoomed In

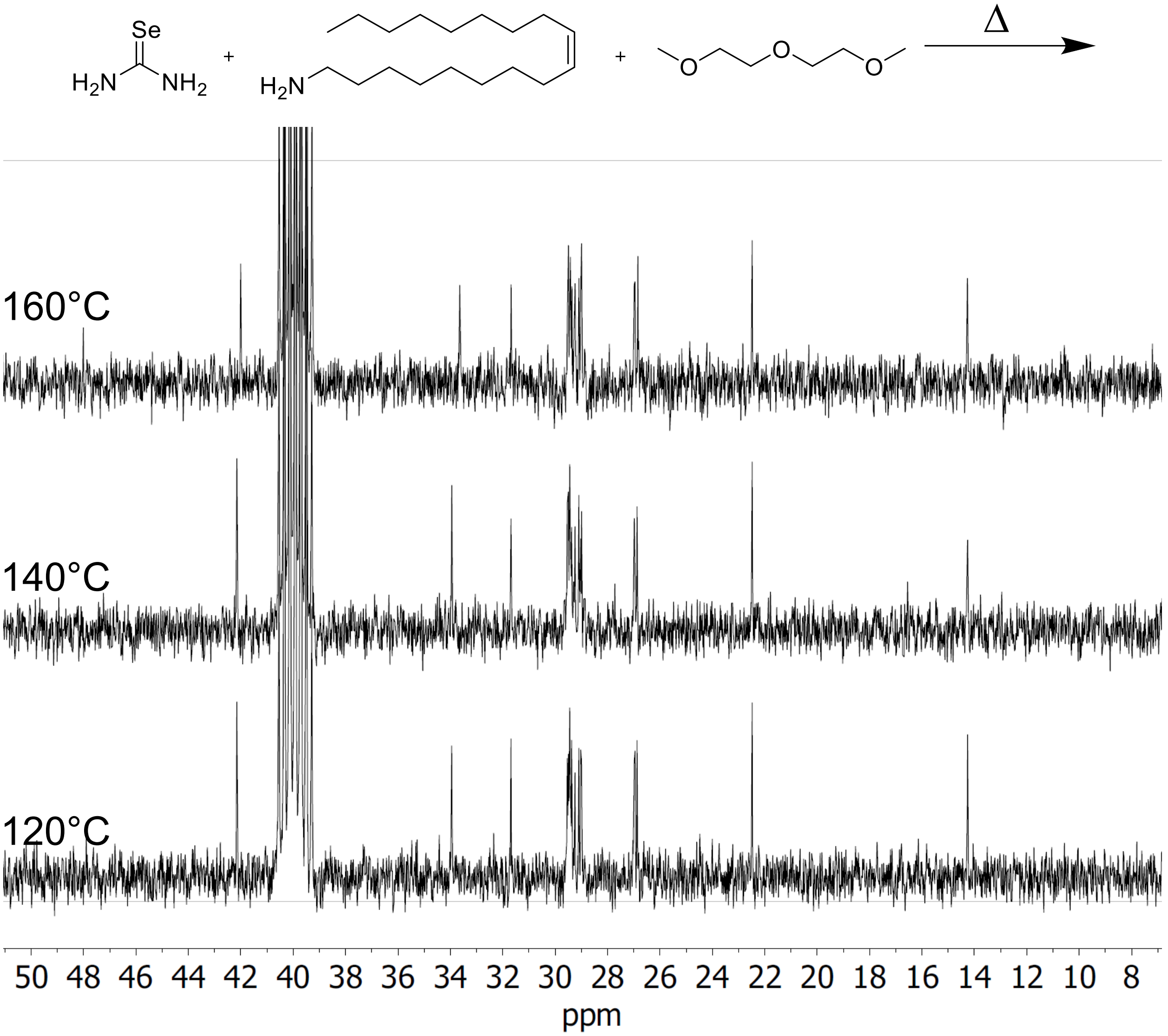

Figure S25. Zoomed in ^{13}C NMR of temperature studies of the reaction between selenourea (0.1 mmol) and oleylamine (0.1 mmol), and diglyme (0.2 mmol). NMR was performed in 600 µL DMSO-d_6.

5.8 ^{1}H NMR Temperature Studies of Oleic Acid and Diglyme Syntheses

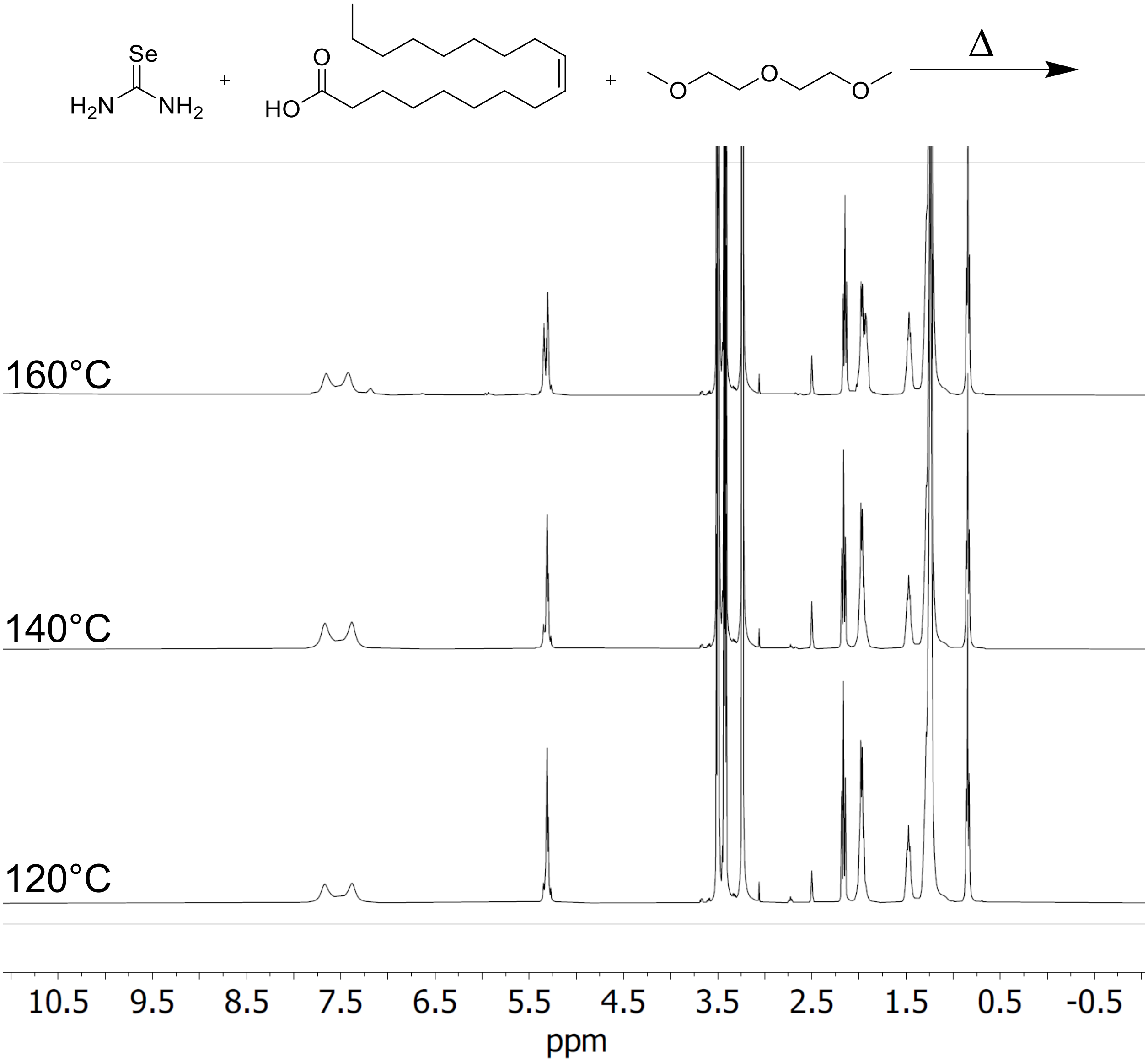

Figure S26. ^{1}H NMR of temperature studies of the reaction between selenourea (0.1 mmol) and oleic acid (0.1 mmol), and diglyme (0.2 mmol). NMR was performed in 600 μL DMSO-d_6.

5.9 ^{13}C NMR Temperature Studies of Oleic Acid and Diglyme Syntheses

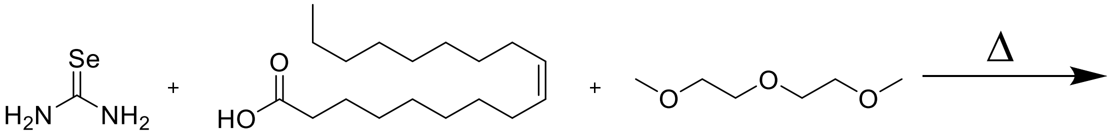

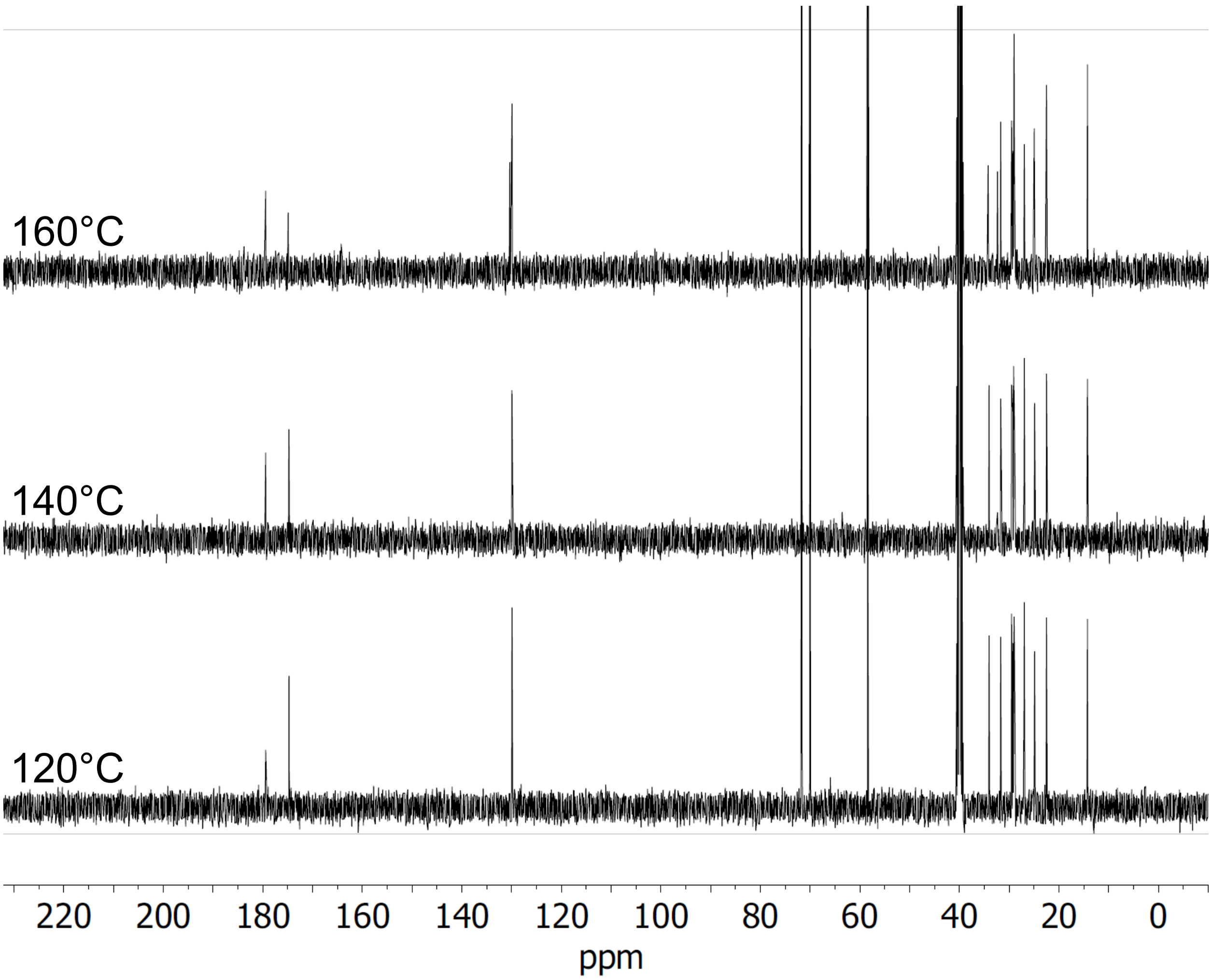

Figure S27. ^{13}C NMR of temperature studies of the reaction between selenourea (0.1 mmol) and oleic acid (0.1 mmol), and diglyme (0.2 mmol). NMR was performed in 600 μL DMSO-d_6.

5.10 ^{13}C NMR Temperature Studies of Oleic Acid and Diglyme Syntheses Zoomed In

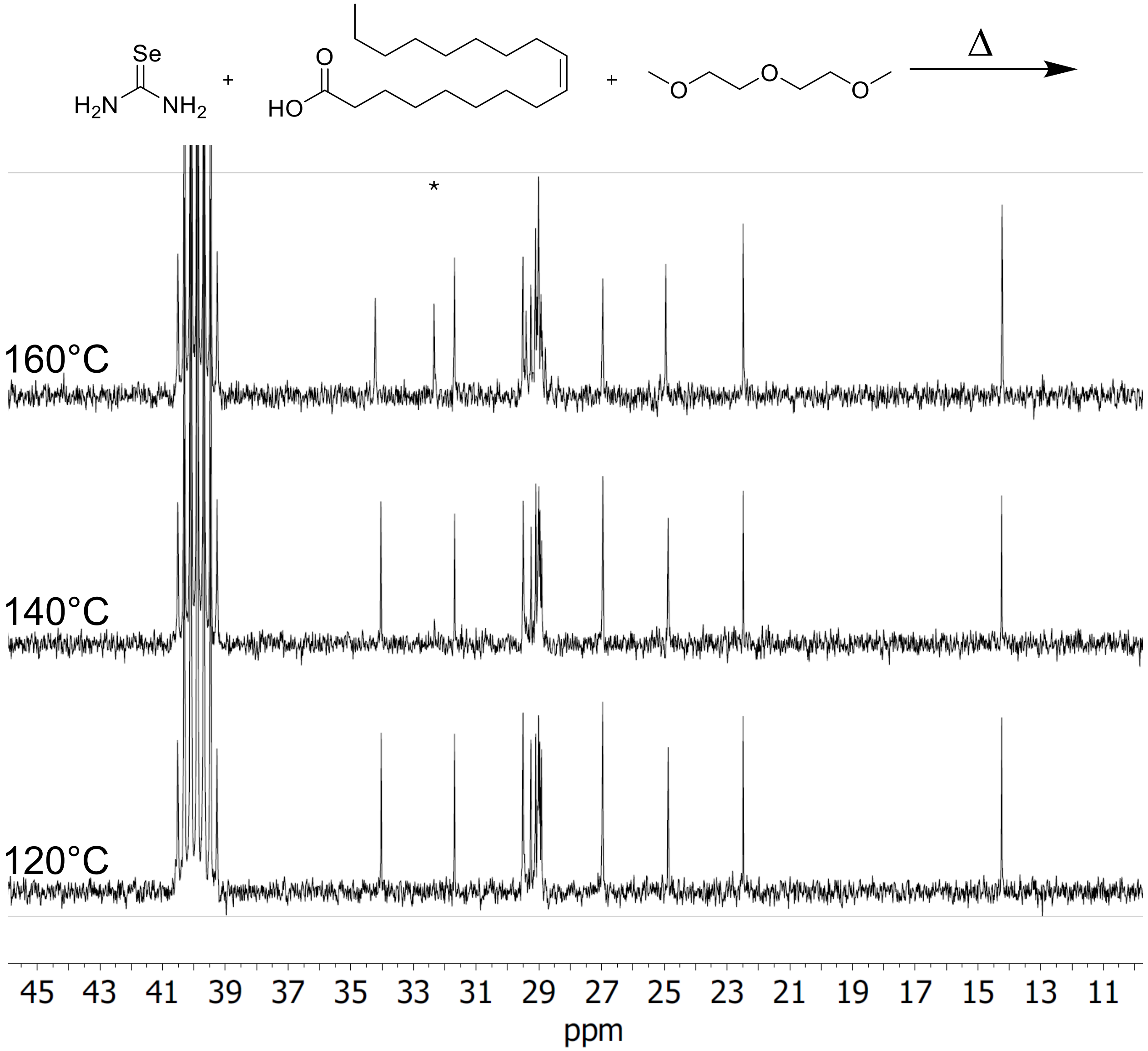

Figure S28. Zoomed in ^{13}C NMR of temperature studies of the reaction between selenourea (0.1 mmol) and oleic acid (0.1 mmol), and diglyme (0.2 mmol). NMR was performed in 600 μL DMSO-d_6. *We attribute the formation of the peak at around δ = 32 ppm to the cis/trans isomerism of oleic acid into elaidic acid.[5]

6. References

(1) Cherin, P.; Unger, P. Refinement of the Crystal Structure of α-Monoclinic Se. *Acta Cryst.* **1972**, *B28* (1), 313–317. https://doi.org/10.1107/S0567740872002249.

(2) Maaninen, T.; Konu, J.; Laitinen, R. S. A Low-Temperature Redetermination of the Monoclinic β-Form of Cyclooctaselenium. *Acta Cryst.* **2004**, *E60* (12), 2235–2237. https://doi.org/10.1107/S1600536804024055.

(3) Foss, O.; Janickis, V. Crystal Structure of γ-Monoclinic Selenium. *J. Chem. Soc., Dalton Trans.* **1980**, No. 4, 624–627. https://doi.org/10.1039/DT9800000624.

(4) Reitzenstein, S.; Rösch, P.; Strehle, M. A.; Berg, D.; Baranska, M.; Schulz, H.; Rudloff, E.; Popp, J. Nondestructive Analysis of Single Rapeseeds by Means of Raman Spectroscopy. *J. Raman Spectrosc.* **2007**, *38* (3), 301–308. https://doi.org/10.1002/JRS.1643.

(5) Iwasaki, T.; Higashikawa, K.; Reddy, V. P.; Ho, W. W. S.; Fujimoto, Y.; Fukase, K.; Terao, J.; Kuniyasu, H.; Kambe, N. Nickel-Butadiene Catalytic System for the Cross-Coupling of Bromoalkanoic Acids with Alkyl Grignard Reagents: A Practical and Versatile Method for Preparing Fatty Acids. *Chem. Eur. J.* **2013**, *19* (9), 2956–2960. https://doi.org/10.1002/CHEM.201204222.
